# GWAS for discovery and replication of genetic loci associated with sudden cardiac arrest in patients with coronary artery disease

**DOI:** 10.1186/1471-2261-11-29

**Published:** 2011-06-10

**Authors:** Bradley E Aouizerat, Eric Vittinghoff, Stacy L Musone, Ludmila Pawlikowska, Pui-Yan Kwok, Jeffrey E Olgin, Zian H Tseng

**Affiliations:** 1 Department of Physiological Nursing, Institute for Human Genetics, University of California, San Francisco, San Francisco, CA 94143 USA; 2 Department of Epidemiology and Biostatistics, University of California, San Francisco, San Francisco, CA 94143 USA; 3 Institute for Human Genetics, University of California, San Francisco, San Francisco, CA 94143 USA; 4 Department of Anesthesia and Perioperative Care, Institute for Human Genetics, University of California, San Francisco, San Francisco, CA 94143 USA; 5 Cardiovascular Research Institute, Institute for Human Genetics, University of California, San Francisco, San Francisco, CA 94143 USA; 6 Section of Cardiac Electrophysiology, Division of Cardiology, Department of Medicine, Institute for Human Genetics, University of California, San Francisco, San Francisco, CA 94143 USA

## Abstract

**Background:**

Epidemiologic evidence suggests a heritable component to risk for sudden cardiac arrest independent of risk for myocardial infarction. Recent candidate gene association studies for community sudden cardiac arrests have focused on a limited number of biological pathways and yielded conflicting results. We sought to identify novel gene associations for sudden cardiac arrest in patients with coronary artery disease by performing a genome-wide association study.

**Methods:**

Tagging SNPs (n = 338,328) spanning the genome were typed in a case-control study comparing 89 patients with coronary artery disease and sudden cardiac arrest due to ventricular tachycardia or ventricular fibrillation to 520 healthy controls.

**Results:**

Fourteen SNPs including 7 SNPs among 7 genes (ACYP2, AP1G2, ESR1, DGES2, GRIA1, KCTD1, ZNF385B) were associated with sudden cardiac arrest (all p < 1.30 × 10^-7^), following Bonferroni correction and adjustment for population substructure, age, and sex; genetic variation in ESR1 (p = 2.62 × 10^-8^; Odds Ratio [OR] = 1.43, 95% confidence interval [CI]:1.277, 1.596) has previously been established as a risk factor for cardiovascular disease. In tandem, the role of 9 genes for monogenic long QT syndrome (LQT1-9) was assessed, yielding evidence of association with CACNA1C (LQT8; p = 3.09 × 10^-4^; OR = 1.18, 95% CI:1.079, 1.290). We also assessed 4 recently published gene associations for sudden cardiac arrest, validating NOS1AP (p = 4.50 × 10^-2^, OR = 1.15, 95% CI:1.003, 1.326), CSMD2 (p = 6.6 × 10^-3^, OR = 2.27, 95% CI:1.681, 2.859), and AGTR1 (p = 3.00 × 10^-3^, OR = 1.13, 95% CI:1.042, 1.215).

**Conclusion:**

We demonstrate 11 gene associations for sudden cardiac arrest due to ventricular tachycardia/ventricular fibrillation in patients with coronary artery disease. Validation studies in independent cohorts and functional studies are required to confirm these associations.

## Background

Sudden cardiac arrest (SCA) remains a major public health problem, causing up to 450,000 deaths per year in the U.S [1]. Approximately 80% of sudden cardiac deaths (SCDs) occur in the setting of coronary artery disease (CAD)[2]. Ventricular tachycardia (VT) or ventricular fibrillation (VF) is the initiating event in the majority of SCAs[3]. Assessment of ejection fraction (EF) remains the only method to identify patients at risk for SCA and primary prevention implantable cardioverter-defibrillator (ICD) implantation, but is both insensitive and nonspecific[4]. Several epidemiologic studies demonstrating that a family history of SCA is an independent risk factor for SCA and primary VF suggest a genetic susceptibility to SCA in the setting of CAD[5-7].

Recent candidate gene association studies for SCA have yielded conflicting results for common variants in the ß2 adrenergic receptor gene[8,9], possibly due to different SCA phenotype definitions - community-based SCA cases as compared to SCA cases due to documented VT/VF in the setting of CAD[10]. Other recent candidate gene studies have yielded associations with SCA for single nucleotide polymorphisms (SNPs) in angiotensin-converting enzyme pathway genes and the transforming growth factor ß-receptor 2 gene, but have yet to be validated[11,12]. A genome wide association study (GWAS) reported SNPs in the NOS1AP gene associated with the prolonged QT interval phenotype, a surrogate of SCA [13]. Association of these NOS1AP SNPs with QT interval has been validated in whites but not blacks from independent cohorts,[13-16] while association with a separate SNP in NOS1AP, not associated with QT interval, has been reported with SCA [17]. A GWAS of the SCA/SCD phenotype has not yet been reported.

The recent report of pilot GWAS of complex traits[18,19] as a precursor to validation studies in larger independent cohorts suggests that such studies could result in cost-effective identification of susceptibility loci of large effect. Thus, the use of rigorous phenotypic criteria for SCA may bolster the power of GWAS to identify risk loci. Accordingly, we performed a pilot GWAS to identify novel genes influencing risk of SCA in a group of patients with a history of myocardial infarction (MI) and aborted SCA with documented VT/VF compared to a healthy control group without SCA or ventricular arrhythmias.

## Methods

The UCSF Committee on Human Research approved all protocols. Informed consent was obtained from all participants for DNA isolation and plasma collection.

### Study Design

Consecutive cases of SCA presenting to University of California, San Francisco (UCSF) Medical Center Emergency Department or inpatient cases of SCA at UCSF Medical Center between January 2000 and June 2008 were screened. SCA was defined as a cardiac arrest with documented sustained monomorphic VT or VF requiring cardioversion or defibrillation, exclusive of torsades de pointes (drug-induced QT prolongation or otherwise). Because we were interested in the most common phenotype of SCA, that occurring the setting of CAD, only patients with a history of MI were included in this study. Although all SCA cases had a history of MI, thirty-six SCA cases occurred in the setting of acute ischemia while 53 SCA cases occurred in the absence of active ischemia. Thus SCA cases consisted of 89 Caucasian non-Hispanic patients with aborted SCD and a history of MI.

The control population originated from three resources [20]. These were randomly selected, healthy Caucasian controls without specific information regarding CAD. Recent large GWAS using shared controls of this type have met with success [20-22]. Sixty unrelated controls employed by the International HapMap Project http://www.HapMap.org were selected from the Coriell Institute for Medical Research http://ccr.coriell.org Human Genetic Data Collection. Two-hundred and sixteen healthy Caucasian controls were derived from a large GWAS of narcolepsy [20]. The remaining 244 controls were healthy Caucasian renal transplant donors from an ongoing study of the genomics of renal transplantation (NIH U19 AI063603). Informed consent was obtained from all participants. Protocols were approved by the local institution review boards at all participating institutions.

### Measurements

Procedures for DNA collection and genotyping using the Genome-Wide SNP Array 6.0 (Affymetrix, Santa Clara, CA) are summarized in the Supplementary Methods (Additional file 1).

### Statistical Analyses

To examine differences in the distribution of demographic factors between cases and controls, we used the unequal-variance t-test for age and a chi-square test for sex. Regression analyses performed to estimate genetic associations controlling for age, sex, and population stratification were conducted in STATAv9.0 (StataCorp, College Station, TX). All other analyses were conducted using HelixTree (GoldenHelix, Bozeman, MT).

The procedures employed to ensure robust genetic association analyses of the 319,222 tagging SNPs[21] including correction for covariates, population substructure[23,24], and batch effects are described in the Supplementary Methods (Additional file 1). The genetic models assessed, procedure for haplotype construction[25] and analyses and significance thresholds established for genome-wide, validation of prior genetic associations (i.e., NOS1AP[13-16], KNG1[11], AGTR1[11]), and novel candidate gene analyses (i.e., monogenic forms of SCA)[26] are also described in the Supplementary Methods (Additional files 1, 2, 3, 4, 5 and 6). The procedures employed to examine the putative function[27,28] of associations are also described in the Supplementary Methods (Additional file 1).

## Results

### Study Population

Compared with controls (Table 1), SCA cases were older (72.8 ± 12.7 versus 52.8 ± 15.5 years; p < 0.0001) and predominantly male (92% versus 52%, p < 0.0001). SCA cases had a mean BMI of 25.8 ± 4.4 kg/m^2 ^and a mean EF of 35.6 ± 14.6%. History of congestive heart failure (CHF) was present in 75.3% of cases; diabetes mellitus in 27.4% of cases, and hypertension in 81.3% of cases. Clinical characteristics were not available for the anonymous healthy controls.

**Table 1:** Demographic Characteristics of SCA Cases and Healthy Controls

			Controls		
			
Characteristics	SCA Cases	All Controls	Narcolepsy	Renal Donor	Hap Map
**N**	88	517	216	241	60
					
Age(years)	72.8 ± 12.7	52.8 ± 15.5	59.0 ± 7.5	42.0 ± 13.8	74.0 ± 7.9
					
Male(%)	92.0	51.8	52.3	51.9	50.0
Congestive Heart Failure(%)	75.3%	N/A			
Ejection Fraction(%)	35.6 ± 14.6	N/A			
Age at index MI(years)	56.6 ± 13.6	N/A			
Hypertension(%)	81.3	N/A			
Diabetes(%)	27.4	N/A			
Current Smoker(%)	10.9	N/A			
Body Mass Index(kg/m^2^)	25.8 ± 4.4	N/A			

### Population Stratification Analysis

The eigenstrat method was utilized to evaluate and adjust for any potential confounding based on subtle differences between groups resulting from differences in ancestry. Genomic control correction factors ranged from 1.027 to 1.086, suggesting a relatively homogenous population. Visual inspection of Q-Q plots of the -log10 P-values generated before and after PCA adjustment, indicated minimal change in the ratio of observed versus expected P-values following PCA correction, additional evidence that population stratification is modest (Additional files 9, 10 and 11).

### GWAS: Identification of Novel Markers for SCA

**Figure 1:**
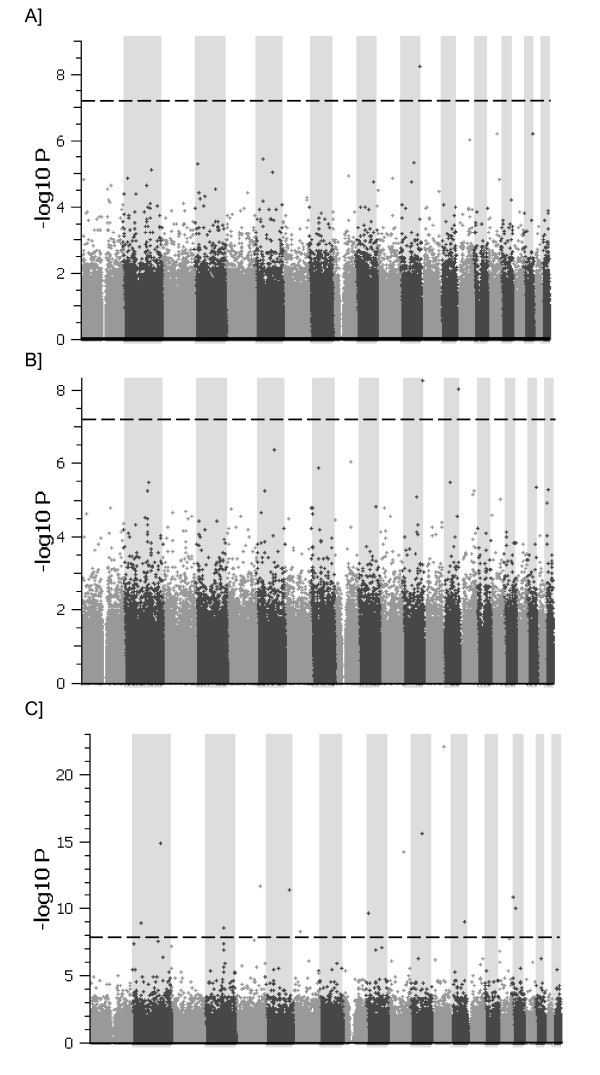
**Summary of genome-wide association scan results for SCA**.

Manhattan plots of the 319,222 tagSNPs employed to interrogate the genomes of the study population are depicted in Figure 1. Fourteen SNPs exceeded the PCA-, age-, and sex-adjusted Bonferroni significance threshold, 7 of which are located in known genes: ACYP2, ZNF385B, GRIA1, ESR1, AP1G2, DEGS2, KCTD1 (Table 2). Of note, genetic variation in one of the genes detected (ESR1) is associated with increased risk of MI in 2 independent populations [29,30]. Given the modest sample size of this study, the top 0.1% (n = 300) PCA-adjusted SNP associations were also tabulated to provide the opportunity for validation in other populations (Additional file 12**: **Table 1). Although many of the 160 genes represented in the top 300 SNP associations are poorly explored *vis a vis *cardiovascular biology, it is noteworthy that one-quarter (n = 41) of these loci are genes with evidence of a role in cardiovascular phenotypes, 2 of which are candidate genes with high suspicion for influencing SCA risk, CACNA1C(LQT8) and NOS3 [31,32]. Q-Q plots of the -log10 P-values of generated after PCA adjustment indicate that all of the SNP associations listed in Table 2 and Additional file 12 (Table 1)deviate considerably from expected P-values following PCA correction, additional evidence that these observations are not likely due to chance (Additional File 13).

**Table 2:** SNPs meeting genome-wide significance threshold after correction for population stratification, age, and sex

Gene	rsID	P_PCA_	P_UNADJ_	OR_PCA-ADJ _(95% CI)	P_PCA-ADJ _	MAF	Call Rate	P_HWE_	Region	Position
ACYP2	rs1559040	1.04E-09	2.11E-06	1.54(1.320,1.787)	3.76e-08	0.111	0.990	1.000000	2p16.2	54201254
ZNF385B	rs16866933	1.45E-15	1.54E-10	1.69(1.475,1.925)	6.15e-14	0.085	0.977	0.059204	2q31.2	180274923
	rs4621553	2.18E-08	5.49E-06	1.40(1.243,1.578)	4.12e-08	0.220	0.995	0.001665	5q22.2	113058063
GRIA1	rs12189362	2.23E-12	9.94E-09	1.50(1.322,1.693)	3.22e-10	0.117	0.977	0.242562	5q33.2	153037741
ESR1	rs2982694	3.91E-12	2.62E-08	1.43(1.277,1.596)	6.96e-10	0.138	0.975	0.160104	6q25.1	152327380
	rs10765792	5.69E-15	4.89E-10	1.54(1.369,1.724)	8.47e-13	0.111	0.980	0.031706	11q21	95506348
	rs7307780	2.27E-16	3.11E-12	1.45(1.327,1.592)	5.03e-15	0.186	0.960	0.141220	12q21.2	74506885
	rs12429889	8.93E-23	7.08E-16	1.64(1.479,1.812)	5.28e-20	0.161	0.954	1.000000	13q22.1	73640323
AP1G2	rs2281680	5.45E-06	1.54E-04	1.38(1.227,1.542)	6.05e-08	0.212	0.982	0.260456	14q11.2	23102910
	rs11624056	8.59E-10	1.20E-06	1.43(1.260,1.615)	3.00e-08	0.110	0.967	0.028261	14q31.3	86576001
DEGS2*	rs7157599	9.14E-09	6.98E-11	1.13(1.082,1.185)	1.30e-07	0.312	0.952	0.830456	14q32.2	99695655
	rs17718586	1.70E-08	7.26E-06	1.53(1.324,1.775)	1.70e-08	0.101	0.964	0.424883	17q24.3	66155784
	rs597503	1.49E-11	1.33E-07	1.45(1.273,1.646)	2.34e-08	0.127	0.959	0.668482	18p11.31	6929947
KCTD1	rs16942421	9.11E-11	3.33E-07	1.68(1.429,1.981)	7.75e-10	0.081	0.975	0.742069	18q11.2	22410423

Unless otherwise indicated, a recessive genetic model fit the data best.

* A dominant genetic model fit the data best.

Abbreviations: **rsID**, **R**eference **S**equence **ID**entifier for a given single nucleotide polymorphism (SNP); **P_PCA_**, p-value for the PCA-corrected test statistic (i.e., Trend Test for the Additive model, Fisher's Exact Test for Dominant and Recessive Models); **P_UNADJ_**, p-value for the unadjusted test statistic (i.e., Correlation); **OR_PCA-ADJ _(95% CI)**, the odds ratio and 95% confidence interval for the test statistic; **P_PCA-ADJ_**, p-value for the test statistic adjusted for PCA, age, and sex; **MAF**, minor allele frequency; **P_HWE_**, p-value for the Hardy-Weinberg test statistic; Region; chromosomal position for the variation; Position, nucleotide position for the variation.

### Prior Gene Associations with SCA

In an attempt to validate previous candidate gene associations reported for SCA, tagSNPs spanning AGTR1, CSMD2, KNG1, and NOS1AP were examined. The tagSNP for each candidate gene with the greatest magnitude of the test statistic is listed in Table 3. We replicated associations for genetic variation in AGTR1 and NOS1AP but not KNG1. Rs263936 in AGTR1 was associated with an increased odds of SCA (adjusted OR = 1.13, 95% CI:1.042-1.215, p = 0.003). According to public databases, the risk allele for SCA in AGTR1 (rs1492099) recently identified[11] is estimated to be in poor linkage disequilibrium (LD) with rs2639365 (r^2 ^= 0.011) in Caucasians. Rs4292933 in NOS1AP was associated with an increased odds of SCA (adjusted OR = 1.15, 95% CI:1.003-1.326, p = 0.045). Though not directly ascertained in our sample, rs4292933 is not in LD with the previously reported NOS1AP risk alleles for QT interval (rs10494366, r^2 ^= 0.091)[13,14] and those SNPs with reported association with SCD (rs12567209 [r^2 ^= 0.245], rs16847548 [r^2 ^= 0.096])[17] in the Caucasian populations sampled in public databases. We also replicated an association with SCA risk for SNP rs1325258 in the CSMD2 gene (adjusted OR = 2.27, 95% CI:1.681, 2.859, p = 0.0061), one of 6 genes meeting significance threshold of p < 10^-4 ^(but not genome-wide significance) in Oregon-SUDS GWAS on SCD but not validated in ARIC/CHS [33].

**Table 3:** Candidate Gene Associations

Gene	rsID	P_PCA_	P_CRUDE_	OR_ADJ _(95% CI)	P_ADJ _	MAF	Call Rate	P_HWE_	Region	Position
**a) Mechanistic candidates from the top 300 SNPs**										
***CACNA1C^†^(LQT8)***	***rs7132154***	***9.29E-05***	***4.72E-04***	***1.18(1.079,1.290)***	***3.09E-04***	***0.262***	***0.992***	***1.000000***	***12p13.33***	***2331484***
***NOS3***	***rs17173656***	***5.41E-05***	***3.09E-04***	***1.13(1.068,1.190)***	***1.53E-05***	***0.102***	***1.000***	***0.786064***	***7q36.1***	***150229592***
**b) Candidate genes for SCA with evidence from the literature**										
AGTR1*	***rs2639365***	***6.16E-04***	***6.20E-04***	***1.13(1.042,1.215)***	***3.00E-03***	***0.059***	***0.998***	***0.317805***	***3q24***	***149783168***
*CSMD2*	*rs1325258*	*2.64E-04*	*5.74E-04*	*2.27(1.681, 2.859)*	*6.10E-03*	*0.095*	*0.995*	*0.768301*	*1p35.1*	*34250168*
KNG1	rs1624230	3.30E-01	2.22E-01	0.99(0.936,1.036)	5.60E-01	0.130	0.974	0.336813	3q27.3	187921629
***NOS1AP^†^***	***rs4292933***	***3.90E-02***	***1.19E-02***	***1.15(1.003,1.326)***	***4.50E-02***	***0.189***	***0.998***	***0.109458***	***1q23.3***	***160417164***
**c) Candidate genes for SCA found in monogenic SCD syndromes (LQT genes)**										
ANK2(LQT4)^†^	rs399754	5.17E-03	4.96E-03	1.14(1.052,1.244)	2.00E-03	0.328	0.964	0.117906	4q26	114311766
CAV3(LQT9)^†^	rs17788626	4.63E-01	4.96E-01	1.00(0.932,1.076)	9.69E-01	0.371	0.998	0.851542	3p25.3	8731423
KCNE1(LQT5)	rs11702354	2.04E-02	2.25E-02	0.96(0.924,1.00)	4.90E-02	0.277	0.957	0.152965	21q22.12	34806395
*KCNE2(LQT6)*	*rs2834455*	*5.09E-04*	*1.28E-03*	*1.08(1.026,1.128)*	*2.00E-03*	*0.145*	*0.987*	*0.565697*	*21q22.11*	*34631628*
KCNJ2(LQT7)^†^	rs12449606	2.27E-02	2.12E-02	1.12(1.024,1.236)	1.40E-02	0.250	0.998	0.625543	17q24.3	65660406
*KCNQ1(LQT1)^†^*	*rs2237877*	*2.21E-02*	*1.49E-02*	*1.17(1.062,1.295)*	*2.00E-03*	*0.226*	*1.000*	*0.433296*	*11p15.5*	*2722810*
SCN5A(LQT3)*	rs6422142	1.57E-03	1.92E-03	1.11(1.044,1.178)	1.00E-03	0.102	1.000	0.786064	3p22.2	38678162

Unless otherwise indicated, an additive genetic model fit the data best.*A dominant genetic model fit the data best.^† ^A recessive genetic model fit the data best. Markers (SNPs) attaining a gene-wide permutation p < 0.01 are rendered in italicized bold font; markers attaining a gene-wide permutation p < 0.05 are rendered in italics. Abbreviations: as in Table 2.

### Long QT Genes

To ascertain if population variation in genes underlying monogenic long QT SCD syndromes is associated with SCA, tagSNPs spanning KCNQ1 (LQT1), SCN5A (LQT3), ANK2 (LQT4), KCNE1 (LQT5), KCNE2 (LQT6), KCNJ2 (LQT7), CACN1AC (LQT8), and CAV3 (LQT9) were examined. The tagSNP for each candidate gene with the greatest magnitude of the test statistic is listed in Table 3. Rs7132154 in CACN1AC (LQT8) was associated with SCA (adjusted OR = 1.18, 95% CI:1.08-1.29, p = 0.0003). Suggestive evidence of association, defined as a gene-wise permutation p<0.05, was observed for rs2834455 in KCNE2 (LQT6) and rs6422142 in KCNQ1 (LQT1).

### Localization of Genetic Associations Using Haplotype Analysis

To evaluate genetic associations that involved more than one tagSNP and to localize the association signals for the genes listed in Tables 2 and 3, haplotype analyses were conducted. The results of the haplotype analyses, using a sliding window approach, are summarized in Table 4. Haplotype analyses for ACYP2, AP1G2, CACNA1C, NOS3, and NOS1AP failed to improve on the initial association signal, and are thus not listed. For ZNF385B, two regions were identified.

**Table 4:** Localization of Associations by Haplotype Analyses

Gene	rs ID	P_PERMUTE_	Haplotypes	Frequency
AGTR1	rs903051	0.0157	AA	0.616
	rs17762633		GA	0.257
			***GC***	0.127
DEGS2	rs2895845	0.0003	GC	0.688
	rs7157599		***GT***	0.312
ESR1	rs3003922	0.0004	GTGTAGG	0.638
	rs7748205		GCATGGG	0.130
	rs3020396		CTACGGG	0.068
	rs3003924		CTACGTA	0.068
	rs1884051		CTACGTG	0.040
	rs2982694		***CTGTATG***	0.020
	rs3020327			
GRIA1	rs7714428	0.0428	ACT	0.338
	rs12189362		CCT	0.300
	rs10041275		CCC	0.241
	rs17115017		CTT	0.105
	rs17115018		***CTC***	0.011
KCTD1	rs10853666	0.0765	CG	0.582
	rs16942421		TG	0.337
			TT	0.068
			***CT***	0.013
ZNF385B	rs9973399	0.0012	***CCT***	0.516
	rs6744352		CCG	0.295
	rs2271759		CAT	0.119
	rs6708300		AAT	0.053
	rs10183243		ACT	0.015
	rs16866933	0.0002	***A***	0.085

Haplotypes rendered in bold and italics differ significantly between cases and controls (p < 0.05). The size of the window is indicated by the number of markers listed for a given block. Haplotype analysis for ACYP2, AP1G2, CACNA1C, NOS3, and NOS1AP failed to improve on the initial association signal. Haplotype analysis was not performed for CSMD2. For ZNF385B, two regions were identified. Abbreviations: **Frequency**, the frequency of each haplotype; **P_PERMUTE_**, p-value for the gene-wise sliding window.

### Assessment of SNP Function

The functional impact of sequence variation in the 14 SNPs meeting genome wide significance and the permuted gene-wise significant SNPs in CACNA1C, NOS3, NOS1AP, CSMD2 and AGTR1 was assessed, revealing that rs7157599 in DEGS2 is a nonsynonymous coding SNP with predicted exonic splicing enhancer effects, residing within a highly-conserved region. The functional impact of the serine to asparagine change (S8N) in DEGS2 is predicted to be benign. Rs12429889, rs17718586, rs16866933 (ZNF385B), rs12189362 (GRIA1), rs2281680 (AP1G2), and rs7132154 (CACNA1C) all reside within highly-conserved regions; the remainder had no predicted impact. Interrogation of the PANTHER database for these 7 genes revealed that each is predicted to have distinct molecular functions and participates in different biological processes including cation transport (ACYP2), nerve-nerve presynaptic transmission (GRIA1), steroid hormone-mediated signaling (ESR1), receptor-mediated endocytosis (AP1G2), cation transport (KCTD1), mRNA transcriptional regulation (ZNF385B), and metabolism (DEGS2).

## Discussion

We demonstrate novel associations in 14 SNPs and SCA due to malignant ventricular arrhythmias in the setting of CAD. Seven of these SNPs were in regions without known genes or regulatory sequences, while the remaining 7 SNPs were located within genes encoding an acylphosphatase (ACYP2), a zinc-finger protein (ZNF385B), a glutamate receptor 1 (GRIA1), an adaptin subunit (AP1G2), a sphingolipid enzyme (DEGS2), a neural potassium channel domain (KCTD1), and an estrogen receptor (ESR1). In tandem, we assessed SNPs in 9 LQT genes and 4 candidate genes with recently reported associations with SCA risk, validating AGTR1, CSMD2, and NOS1AP but not KNG1. Although not meeting genome-wide significance, notable among the top 0.1% (n = 300) SNP associations, which included 160 genes, were 1 SNP in CACNA1C (LQT8) and 3 SNPs in NOS3. SNP associations in both of these genes met permuted gene-wise significance and both genes have strong mechanistic rationale as candidate genes for SCA.

Although we included a modest number of cases, these were rigorously phenotyped, all with confirmed CAD and aborted SCA due to documented VT/VF, and were compared to healthy controls without SCD. The requirement for VT/VF in all SCA cases maximizes the power of the study by avoiding the possibility of association with other spurious causes of out-of-hospital sudden death (i.e., pulmonary embolus, bradycardia, acute heart failure). SCD as defined in prior studies[33], a sudden pulseless condition of cardiac origin in a previously stable individual,[17] is more heterogeneous and necessarily includes many noncardiac and/or nonarrhythmic causes of sudden death misclassified as SCD [10]. Because we used shared controls, principal components analysis was employed to exclude the possibility of population stratification as a cause of spurious association. Although we did not test our SNP associations in a validation cohort, we found associations in genes with high prior likelihood of mediating SCA risk in the setting of CAD (ESR1, CACNA1C, NOS3) and our replication of associations with SCA risk for genetic variation in 3 previously reported genes (NOS1AP, CSMD2, AGTR1) substantiates the value of this study. Moreover, our conservative approaches *vis a vis *analytic methodology (i.e., PCA-correction, QC criteria) and significance thresholds (i.e., Bonferroni correction, permutation) suggests that the findings are robust. In contrast to the recent GWAS on SCD which did not discover any genetic associations meeting genome-wide significance[33], we report 7 SNP associations surpassing this stringent threshold.

Little is known about the potential roles of ACYP2, ZNF385B, GRIA1, AP1G2, DEGS2, and KCTD1 in cardiovascular physiology. A large body of evidence supports the impact of estrogen receptor alpha (ESR1), which regulates the expression of multiple genes after activation by estrogen, in cardiovascular disease in both men and women. A higher risk of ischemic cardiac event has been reported for 2 separate nonsynonymous SNPs in ESR1 [29,30]. In postmenopausal women, carriers of ESR1 haplotype 1 also have a higher risk of death from such events, although it is unknown whether these represent sudden deaths due to ventricular arrhythmias. Rs2982694 is not predicted to result in functional change in ESR1 and is not in LD with the prior studied ESR1 SNPs (rs2234693 [r^2 ^= 0.000], rs9340799 [r^2 ^= 0.000]) in Caucasians. We had too few women to study them separately; however, adjustment for sex in the regression analyses did not appreciably change our results (data not shown). Whether our results represent association of genetic variation in ESR1 with the intermediate phenotype CAD rather than SCA requires further investigation.

Because of prior evidence, our threshold for significance for SNPs in the previously studied genes NOS1AP, CSMD2, AGTR1, and KNG1 was p<0.05. Association of SNPs in NOS1AP has been consistently reported with the intermediate phenotype of prolonged QT intervals in Caucasians as well as SCD [13-17]. Rs4292933 in NOS1AP in the present study, appears to be distinct from those studied previously (i.e., they are not in LD). We also found an association with SCA risk in the setting of CAD for 2 SNPs in CSMD2, validating one of top gene associations in the Oregon-SUDS discovery phase of the recent GWAS, but which was not validated in ARIC or CHS [13]. This may reflect the fact that our SCA cohort is more similar to the Oregon-SUDS cohort, both of which required documented CAD, than the validation ARIC and CHS cohorts which included more heterogeneous SCD cases, thus restricting the power to replicate associations with SCA/SCD in the setting of CAD. Among reported associations with SCA for SNPs in genes in the angiotensin-converting enzyme-associated pathway[11], we replicated the role of genetic variation in AGTR1, but not KNG1.

We separately assessed tagSNPs in 9 long QT genes causing forms of the monogenic long QT syndrome which carry high risk for SCD and thus high prior rationale for association with SCA. In addition to rs7132154 in CACNA1C(LQT8) which was among the top 300 SNP associations and met gene-wise permuted significance p<0.01, rs6422142 in KCNQ1(LQT1) and rs2834455 in KCNE1(LQT6) were associated with increased odds of SCA at the gene-wise permutation threshold p<0.05. These SNPs are not predicted to have functional impact and are presumed to be in LD with the causative variant(s).

Because we employed highly conservative Bonferroni correction to arrive at the 14 SNPs meeting genome-wide significance and limited power to detect additive effects due to the sample size, we may have missed some associations with SCA; we thus report the top 300 PCA-adjusted SNP associations to provide the opportunity for validation in other populations. These SNPs reside in 160 known genes, 41 of which have been reported to play a role in cardiovascular physiology and/or are expressed in the heart. Of these, we found 1 SNP (rs7132154) in CACNA1C and 3 SNPs (rs17173656, rs17173658, rs10264084) in NOS3 that met gene-wise permutation significance thresholds; these two genes have key roles in arrhythmogenesis. Mutations in CACNA1C, which encodes the alpha 1C subunit of the voltage-dependent, L-type calcium channel, lead to lack of voltage-dependent inactivation and prolonged inward calcium current and cause Timothy syndrome (LQT8), a multisystem disorder characterized by long QT intervals and cardiac, hand/foot, facial, and neurodevelopmental features; malignant VT is present in 80% and are the leading cause of death [32]. Functional changes in caveolar-localized endothelial NOS have been identified post-MI and NOS3-deficient mouse models have demonstrated a higher incidence of spontaneous and inducible VT/VF [31]. Intriguingly, NOS3 regulates the activity of L-type calcium channels (CACNA1C comprises the pore-forming subunit) via the ß-adrenergic system, impacting contractility and excitability [34]. Reduction or elimination of NOS3-produced nitric oxide, which by itself attenuates β-adrenergic stimulation of L-type calcium channel currents[34], causes higher influx of calcium ions which can lead to two potent triggers of ventricular arrhythmias: early and delayed afterdepolarizations [35]. Thus, although NOS1AP does not directly bind NOS3, both directly affect L-type calcium current [36]. Therefore, taken together these CACNA1C, NOS3, and NOS1AP SNPs may represent a key electrophysiologic pathway (L-type calcium current) enriched for risk genotypes for SCA. Although none of the SNPs in CACNA1C and NOS3 are predicted to have functional impact, rs7132154 in CACNA1C is in a highly-conserved region; presumably these SNPs are in LD with the causal variant(s). Further study is necessary to fine-map these loci, identify causative variants, and elucidate their impact on CACNA1C and NOS3 function or levels, signaling, L-type calcium current, and whether they ultimately affect development of ventricular arrhythmias.

There are notable limitations to our study. We examined tagSNPs in genes covered by the DNA SNP array, rarer polymorphisms and insertion/deletion variants were not assessed by our study. Stringent control for the number of statistical tests substantially reduces power, in particular given the limited number of SCA cases, such that some associations in the examined SNPs may have been missed. However, these same stringent procedures increase the confidence that the association signals detected represent true positive results. Furthermore, because only Caucasian cases and controls were examined, further studies are needed to determine whether these findings can be generalized to SCA in other ethnicities. Healthy controls were used, thus a confounding association with CAD or other intermediate phenotypes such as CHF or hypertension cannot be ruled out. Finally and most importantly, the possibility that these associations are due to chance should be considered, thus validation of these findings in independent populations is critical.

## Conclusion

In summary, the results of this pilot GWAS provide novel evidence for increased arrhythmic SCA risk in the setting of CAD due to genetic variation in six genes without prior evidence of cardiovascular effect (ACYP2, ZNF385B, GRIA1, AP1G2, DEGS2, KCTD1) and in a number of genes with strong prior evidence of arrhythmic and/or cardiovascular effects (ESR1, CACNA1C, NOS3, NOS1AP, CSMD2, AGTR1, KCNQ1, KCNE1). These findings contribute to accumulating evidence for the influence of genetic variation in risk of SCA due to malignant ventricular arrhythmias in CAD patients. If these results are validated, further investigation of these variants in the development of ventricular arrhythmias is warranted.

## Abbreviations

(ACYP2): Acylphosphatase; (AP1G2): Adaptin subunit; (AGTR1): Angiotensin II receptor, type 1; (CACNA1C): Calcium channel, voltage-dependent, L type, alpha 1C subunit; (CI): Confidence interval; (CHF): Congestive heart failure; (CAD): Coronary artery disease; (CSMD2): CUB and sushi domain-containing protein 2; (EF): Ejection fraction; (ESR1): Estrogen receptor; (GWAS): Genome wide association study; (GRIA1): Glutamate receptor 1; (ICD): Implantable cardioverter-defibrillator; (LD): Linkage disequilibrium; (MI): Myocardial infarction; (NOS1AP): Nitric oxide synthase 1 adaptor protein; (NOS3): Nitric oxide synthase 3; (OR): Odds ratio; (KCNE1): Potassium voltage-gated channel, Isk-related family, member 1,; (KCNQ1): Potassium voltage-gated channel, KQT-like subfamily, member 1; (PCA): Principal component analysis; (SNP): Single nucleotide polymorphism; (SCA): Sudden cardiac arrest; (SCD): Sudden cardiac death; (DEGS2): Sphingolipid enzyme; (KCTD1): Neural potassium channel domain; (VF): Ventricular fibrillation; (VT): Ventricular tachycardia; (US): United States; (UCSF): University of California, San Francisco; (ZNF385B): Zinc-finger protein.

## Competing interests

None of the authors claim any competing interests with respect to the findings presented herein.

## Authors' contributions

All authors read and approved the final manuscript. BAE conceived of the study, designed the study, provided funds to support the study, performed the statistical analyses, and wrote the manuscript. EV performed statistical analyses, and assisted in preparation and revision of the manuscript. SLM performed genotypic data collection. LP assisted in the design of the study, assisted in genotypic data collection, and assisted in preparation of the manuscript. PYK assisted in the design of the study and provided resources to support genotypic data collection. JEO assisted in the design of the study, provided resources to support genotypic data collection, and assisted in preparation of the manuscript. ZHT conceived of the study, designed the study, provided funds to support the study, recruited participants for the study, and wrote the manuscript.

## Financial support

This research was funded by grants from the National Center for Research Resources (NCRR), a component of the National Institutes of Health (NIH) and NIH Roadmap for Medical Research (KL2 RR024130) to BEA and ZHT, and the National Heart, Lung, and Blood Institute/National Institutes of Health (NHLBI/NIH R01 HL102090-01) to ZHT.

## Pre-publication history

The pre-publication history for this paper can be accessed here:

http://www.biomedcentral.com/1471-2261/11/29/prepub

## Supplementary Material

Additional file 1 **Supplemental Methods**. Supplementary Methods Click here for file

Additional file 2 **Empirically-determined quality control thresholds for SNP Call Rate and Hardy-Weinberg Equilibrium**. In Panel A, the region of the point of inflection in SNP call rate was rendered with call rate on the Y axis and SNPs ordered by call rate on the X-axis. In Panel B, HWE p-values are plotted along the Y-axis with SNPs ordered by HWE p-value on the X-axis, the inflection point (p < 0.00015) indicated. Click here for file

Additional file 3 **Association localization plots for ACYP2 verifying minimal overlap in tagSNP selection**. Results for tagSNPs used in the discovery phase (adjusted for principal components) are presented as circles. Negative LOG p-values are provided on the Y axis. The × axis corresponds to the locations of SNPs. The p-value obtained with the most highly associated SNP is indicated with a green arrow. The LD relationship (R2) of the tagSNPs analyzed in the pilot GWAS are shown below the graph with shared variance between tagSNP pairs ranging from gold (low shared variance) to red (complete shared variance). Click here for file

Additional file 4 **Association localization plots for ZNF385B verifying minimal overlap in tagSNP selection**. In Panel A, the region of the point of inflection in SNP call rate was rendered with call rate on the Y axis and SNPs ordered by call rate on the X-axis. In Panel B, HWE p-values are plotted along the Y-axis with SNPs ordered by HWE p-value on the X-axis, the inflection point (p < 0.00015) indicated. Click here for file

Additional file 5 **Association localization plots for GRIA1 verifying minimal overlap in tagSNP selection**. Results for tagSNPs used in the discovery phase (adjusted for principal components) are presented as circles. Negative LOG p-values are provided on the Y axis. The × axis corresponds to the locations of SNPs. The p-value obtained with the most highly associated SNP is indicated with a green arrow. The LD relationship (R2) of the tagSNPs analyzed in the pilot GWAS are shown below the graph with shared variance between tagSNP pairs ranging from gold (low shared variance) to red (complete shared variance). Click here for file

Additional file 6 **Association localization plots for ESR1 verifying minimal overlap in tagSNP selection**. Results for tagSNPs used in the discovery phase (adjusted for principal components) are presented as circles. Negative LOG p-values are provided on the Y axis. The × axis corresponds to the locations of SNPs. The p-value obtained with the most highly associated SNP is indicated with a green arrow. The LD relationship (R2) of the tagSNPs analyzed in the pilot GWAS are shown below the graph with shared variance between tagSNP pairs ranging from gold (low shared variance) to red (complete shared variance). Click here for file

Additional file 7 **Association localization plots for DEGS2 verifying minimal overlap in tagSNP selection**. Results for tagSNPs used in the discovery phase (adjusted for principal components) are presented as circles. Negative LOG p-values are provided on the Y axis. The × axis corresponds to the locations of SNPs. The p-value obtained with the most highly associated SNP is indicated with a green arrow. The LD relationship (R2) of the tagSNPs analyzed in the pilot GWAS are shown below the graph with shared variance between tagSNP pairs ranging from gold (low shared variance) to red (complete shared variance). Click here for file

Additional file 8 **Association localization plots for KCTD1 verifying minimal overlap in tagSNP selection**. Results for tagSNPs used in the discovery phase (adjusted for principal components) are presented as circles. Negative LOG p-values are provided on the Y axis. The × axis corresponds to the locations of SNPs. The p-value obtained with the most highly associated SNP is indicated with a green arrow. The LD relationship (R2) of the tagSNPs analyzed in the pilot GWAS are shown below the graph with shared variance between tagSNP pairs ranging from gold (low shared variance) to red (complete shared variance). Click here for file

Additional file 9 **GWAS Q-Q plots for the additive model of component control groups**. Q-Q plots of the negative log10 P values for crude (Panels A, C, and E) and PCA-corrected (Panels B, D, and F) correlation tests for genome-wide association across the genome are shown for the Renal Transplant Donor Controls as compared to the Narcolepsy Controls (Panels A and D), HapMap Controls as compared to the Narcolepsy Controls (Panels B and D), and the HapMap Controls as compared to the Renal Transplant Donor Controls (Panels C and F). Click here for file

Additional file 10 **GWAS Q-Q plots for the dominant model of component control groups**. Q-Q plots of the negative log10 P values for crude (Panels A, C, and E) and PCA-corrected (Panels B, D, and F) correlation tests for genome-wide association across the genome are shown for the Renal Transplant Donor Controls as compared to the Narcolepsy Controls (Panels A and D), HapMap Controls as compared to the Narcolepsy Controls (Panels B and D), and the HapMap Controls as compared to the Renal Transplant Donor Controls (Panels C and F). Click here for file

Additional file 11 **GWAS Q-Q plots for the recessive model of component control groups**. Q-Q plots of the negative log10 P values for crude (Panels A, C, and E) and PCA-corrected (Panels B, D, and F) correlation tests for genome-wide association across the genome are shown for the Renal Transplant Donor Controls as compared to the Narcolepsy Controls (Panels A and D), HapMap Controls as compared to the Narcolepsy Controls (Panels B and D), and the HapMap Controls as compared to the Renal Transplant Donor Controls (Panels C and F). Click here for file

Additional file 12 **List of top 300 SNPs identified after correction for population stratification**. SNPs also listed in Table 2 (significant after Bonferroni correction) are boxed for ease of identification. Genes with evidence of a role in cardiovascular phenotype are rendered in italics. Genes that are mechanistically recognized candidates for SCA are rendered in italicized bold font. Unless otherwise indicated, a recessive genetic model fit the data best. Abbreviations: rsID, Reference Sequence IDentifier for a given single nucleotide polymorphism (SNP); PPCA, p-value for the PCA-corrected test statistic (i.e., Armitage Trend Test for the Additive model, Fisher's Exact Test for Dominant and Recessive Models); PUNADJ, p-value for the unadjusted test statistic (i.e., Correlation Test); ORPCA-ADJ (95% CI), the odds ratio and 95% confidence interval for the test statistic; PPCA-ADJ, p-value for the test statistic adjusted for PCA, age, and sex; MAF, minor allele frequency; PHWE, p-value for the Hardy-Weinberg test statistic; Region; chromosome and band position for the variation; Position, nucleotide position for the variation. Click here for file

Additional file 13 **GWAS Q-Q plots featuring location of candidate SNPs**. Q-Q plots of the negative log10 P values for PCA-corrected correlation tests for genome-wide association across the genome are shown for the additive (panel A), Dominant (panel B), and Recessive (panel C) models. Those SNPs associations that reached genome-wide significant (Bonferroni-p <0.05) are colored in red. Click here for file
