# Supplementary material for: GWAS for discovery and replication of genetic loci associated with sudden cardiac arrest in patients with coronary artery disease
Source: BMC Cardiovasc Disord. 2011 Jun 10;11:29. doi: 10.1186/1471-2261-11-29 (PMC3141757; doi:10.1186/1471-2261-11-29)
Supplement: Additional file 1 — Supplemental Methods. Supplementary Methods [file 1471-2261-11-29-S1.DOC]

### Additional file 1

**Title: Supplemental Methods**

# Description: Supplementary Methods

## Measurements

Blood collection and genotyping: Blood samples were obtained by venipuncture and genomic DNA was extracted from peripheral blood lymphocytes (Invitrogen, Carlsbad, CA) for the SCA cases. Genotyping was performed blinded to clinical status; positive and negative controls were included. DNA samples were quantitated with a Nanodrop Spectrophotometer (ND-1000) and normalized to a concentration of 50 ng/μL (diluted in 10 mM Tris/1 mM EDTA).

Samples were genotyped using the Genome-Wide SNP Array 6.0 (Affymetrix, Santa Clara, CA) and processed according to the standard Affymetrix automated protocol, which is summarized in the Supplementary Methods. In brief, this involved fragmentation, whole genome amplification, precipitation, re-suspension in hybridization buffer and hybridization to the Genome-Wide SNP Array 6.0. After hybridization the arrays were processed and imaged on an Affymetrix GeneChip® Scanner. Genotype data for the cases and controls were obtained using the same protocols in the same laboratory.

The electronic hybridization data was saved as DAT and CEL files for all samples (n=608). The CAB files for the images were used to transfer the data into GCOS v1.0 for subsequent use of the data in G-TYPE v4.1 software. Genotype calls were assigned using the Birdseed-dev algorithm for Affy 6.0 (Affymetrix Power Tools apt-1.8.5). Although the cases and the three sources for controls were genotyped separately as the controls were contributed from existing data collected in tandem to the controls, the Birdseed-dev algorithm was applied to the merged dataset of all, cases and controls. Initially, 907,811 SNPs were available for analysis. For SNPs with association signals of interest (i.e., genome-wide significant associations, top 300 association signals), probe cluster plots were visually examined to verify appropriate clustering.

## Statistical Analyses

Table SNP Selection for Genome Wide Association: In order to ensure robust genetic association analyses, we performed rigorous and conservative quality control filtering of both samples and SNPs as recommended by the Wellcome Trust Case Control Consortium.[1] The first step was to exclude samples with poor quality genome-wide data; sample call rate was plotted for the entire sample (n=608) and the empirically determined threshold defined as the distribution’s point of inflection was set at less than 93% of SNPs successfully genotyped. One case and three controls were thus excluded. The second step was to exclude SNPs with poor quality data. SNP call rate was plotted for the entire sample (n=604) and the empirically determined threshold defined as the distribution’s point of inflection was set at 95% (**Additional file 7, panel A**), resulting in the exclusion of 54,590 SNPs of poor quality. Given the goal of identifying common genetic risk alleles for SCA, 166,748 SNPs with a minor allele frequency (MAF) less than 5% were excluded from subsequent analyses. Finally, in order to reduce false positive associations, we filtered out SNPs deviating from empirically determined thresholds of Hardy-Weinberg expectations (HWE) in the control group. This threshold was determined by Q-Q plot with SNPs yielding a HWE p-value less than 0.00015 excluded from further analysis (**Additional file 7, panel B**), which resulted in the removal of 26,416 SNPs. Of note, this HWE threshold is considerably more conservative than a Bonferroni correction (p<10^-7^). A total of 660,057 SNPs passed all quality control filters and were included in tagging SNP selection.

Tagging SNP Selection: In order to reduce redundancy in SNP data due to presence of markers in high linkage disequilibrium (LD) in the dataset, tagging SNPs (tagSNPs) where selected. The tagSNP selection algorithm established by Carlson and colleagues was employed[2]; we sought representative tagSNPs in LD > 0.80 with a maximal search window of 50 SNPs adjacent to the candidate tagSNP. This reduced the number of SNPs for association analysis from 660,057 to 319,222. Non-redundancy in tagSNP selection was verified by association localization plots for the 6 (i.e., ACYP2, ZNF385B, GRIA1, ESR1, DEGS2, KCTD1) of the 7 genetic associations attaining genome-wide significance thresholds that map to known genes and that are identified in **Table 2**. An association localization plot for the 7^th^ locus, AP1G2, was not rendered due to the fact that only one additional tag SNP mapped to the region and was assessed in the subsequent haplotype analyses (**Table 4**). The six association localization plots can be found in **Additional files 8-19** for ACYP2, ZNF385B, GRIA1, ESR1, DEGS2, and KCTD1, respectively.

Controlling for Population Substructure: Homogeneity in ancestry between cases and controls was verified by cluster and principal component analysis (PCA).[3] To investigate other biases that could be introduced by using controls shared with other studies such as batch effects due to differences in instrumentation[4], we assessed the potential effect of substructure with the genomic-control method[5] and with PCA, as implemented in HelixTree (GoldenHelix, Bozeman, MT). Briefly, the number of principal components were sought which minimized the value of the genome control test statistic (λGC). Two principal components were selected under the additive (λGC=1.027) and recessive (λGC=1.086) models; three principal components were selected under the dominant model (λGC=1.058). Population substructure was found to be modest, with no discernible batch effects; PCA-adjusted tests of association showed minimal variation as compared with unadjusted tests (data not shown).

Genetic models: For association tests, three genetic models were assessed for each SNP: additive, dominant, and recessive. Barring trivial improvements (delta<10%) the genetic model that best fit the data, defined as maximizing the significance of the p-value, was selected for each SNP. PCA-corrected correlation/trend tests were used to compare the three genetic models. Unadjusted associations were also estimated: the Armitage Test for the additive model and Fisher’s Exact Test for both dominant and recessive models. Markers of interest were further adjusted for PCA, age, and sex by logistic regression; adjusted odds ratios and 95% confidence intervals (95% CI) for the SNP were also calculated. Regression analyses were performed with Intercooled Stata 9.2 for Windows.

Candidate Gene Analyses of SNPs with Recently Reported Association with SCA: HWE was assessed by the chi-square exact test. Measures of linkage disequilibrium, D’ and r2, were computed from the case and control genotypes and heat maps of pairwise D’ values were rendered for visual inspection. For association tests, three genetic models were assessed for each SNP: additive, dominant, and recessive. Barring trivial improvements (delta<10%) the genetic model that best fit the data, by maximizing the significance of the p-value, was selected for each SNP. Both unadjusted and adjusted associations were calculated; linear regression was used to control for PCA-corrected outcome and genotype data, age, and sex. Gene-wise permutation tests were used to protect the type-I error rate against inflation due to testing of multiple SNPs within each gene. To account for multiple comparisons, case-control status was permuted 10,000 times to determine the likelihood that our findings were due to chance.

Haplotype Analyses: For those SNPs within genes attaining the required significance threshold (described below), haplotype analyses were conducted in order to localize the association signal within the gene and to determine if haplotypes improved the strength of the association with SCA. A sliding-window of markers was used, beginning with 2 SNP markers and was serially increased by one marker. Barring trivial improvements (delta<10%) the number of markers (i.e., single SNPs, multi-SNP haplotypes) was selected that best fit the data by maximizing the significance of the p-value. The expectation-maximization (EM) method was employed to estimate haplotype frequencies.[6] A haplotype frequency threshold of >0.01 was used to reduce the variables considered in association tests; a maximum of 50 EM iterations and a convergence tolerance of 0.00001 were employed. Significant differences in the distribution of haplotypes between cases and controls were determined by Chi-square test with gene-wide permutation applied to the results as described in the preceding sub-section.

Significance Thresholds: The threshold for statistical significance for the GWAS was established using the Bonferroni correction (p=0.05/319,222 = 1.57e-7). The analysis of SNPs selected to replicate the association of candidate genes with SCA as reported in recent investigations (NOS1AP[7-11], KNG1[12], AGTR1[12], CSMD2[13]) was required to attain a gene-wise permutation p-value of p<0.05. A more conservative approach (i.e., gene-wise permutation p<0.01) was adopted for the assessment of the candidate genes causing monogenic forms of SCA (i.e., ANK2 [LQT4], CACNA1C [LQT8], CAV3 [LQT9], KCNE1 [LQT5], KCNE2 [LQT6], KCNJ2 [LQT7], KCNQ1 [LQT1], SCN5A [LQT3]).[14] Of note, tagSNPs for KCNH2 [LQT2] were not represented in the dataset. For tagSNPs listed in Tables 2-4 and Table 1 that met the above listed significance thresholds, a post hoc test for informative missingness was performed where markers displaying informative missingness between cases and controls p<0.001 were excluded from further analysis. None of the SNPs met this exclusion criterion..

Assessment of SNP Function: Potential functional roles of SNPs associated with SCA were examined using PUPASuite 2.0,[15] a comprehensive search engine for functional effects (e.g., non-synonymous changes, altered transcription factor binding sites, exonic splicing enhancing/silencing, microRNA target alterations) (http://pupasuite.bioinfo.cipf.es). Linkage disequilibrium estimates for candidate gene for SCA/SCD previously reported in the literature (i.e., AGTR1, KNG1, NOS1AP, CSMD2) were sought by interrogation of public databases (i.e., HapMap [http://www.HapMap.org], SeattleSNP [http://pga.gs.washington.edu]). For those SNPs resulting in alterations in the amino acid sequence, the functional impact of the change was explored using PolyPhen (Polymorphism Phenotyping; http://genetics.bwh.harvard.edu/pph/), a tool which predicts possible impact of an amino acid substitution on the structure and function of a human protein using empirical rules applied to the sequence, in addition to phylogenetic and structural information characterizing the substitution.[16]

To ascertain if the genes identified by the GWAS represent pathways expected to be altered in the pathogenesis of SCA (e.g., beta adrenergic receptor signaling pathway), the PANTHER (Protein ANalysis THrough Evolutionary Relationships) Classification System was applied to these genes (http://www.pantherdb.org/). PANTHER is a unique resource that classifies genes by their functions, using published scientific experimental evidence and evolutionary relationships to predict function even in the absence of direct experimental evidence.

## REFERENCES

1. Wellcome Trust Case Control C: **Genome-wide association study of 14,000 cases of seven common diseases and 3,000 shared controls.** *Nature* 2007, **447:**661-678.

2. Carlson CS, Heagerty PJ, Nord AS, Pritchard DK, Ranchalis J, Boguch JM, Duan H, Hatsukami TS, Schwartz SM, Rieder MJ, et al: **TagSNP evaluation for the association of 42 inflammation loci and vascular disease: evidence of IL6, FGB, ALOX5, NFKBIA, and IL4R loci effects.** *Hum Genet* 2007, **121:**65-75.

3. Price AL, Patterson NJ, Plenge RM, Weinblatt ME, Shadick NA, Reich D: **Principal components analysis corrects for stratification in genome-wide association studies.** *Nat Genet* 2006, **38:**904-909.

4. Plenge RM, Padyukov L, Remmers EF, Purcell S, Lee AT, Karlson EW, Wolfe F, Kastner DL, Alfredsson L, Altshuler D, et al: **Replication of putative candidate-gene associations with rheumatoid arthritis in >4,000 samples from North America and Sweden: association of susceptibility with PTPN22, CTLA4, and PADI4.** *Am J Hum Genet* 2005, **77:**1044-1060.

5. Devlin B, Roeder K, Wasserman L: **Genomic control, a new approach to genetic-based association studies.** *Theor Popul Biol* 2001, **60:**155-166.

6. Excoffier L, Slatkin M: **Maximum-likelihood estimation of molecular haplotype frequencies in a diploid population.** *Mol Biol Evol* 1995, **12:**921-927.

7. Aarnoudse AJ, Newton-Cheh C, de Bakker PI, Straus SM, Kors JA, Hofman A, Uitterlinden AG, Witteman JC, Stricker BH: **Common NOS1AP variants are associated with a prolonged QTc interval in the Rotterdam Study.** *Circulation* 2007, **116:**10-16.

8. Arking DE, Pfeufer A, Post W, Kao WH, Newton-Cheh C, Ikeda M, West K, Kashuk C, Akyol M, Perz S, et al: **A common genetic variant in the NOS1 regulator NOS1AP modulates cardiac repolarization.** *Nat Genet* 2006, **38:**644-651.

9. Eijgelsheim M, Aarnoudse AL, Rivadeneira F, Kors JA, Witteman JC, Hofman A, van Duijn CM, Uitterlinden AG, Stricker BH: **Identification of a common variant at the NOS1AP locus strongly associated to QT-interval duration.** *Hum Mol Genet* 2009, **18:**347-357.

10. Lehtinen AB, Newton-Cheh C, Ziegler JT, Langefeld CD, Freedman BI, Daniel KR, Herrington DM, Bowden DW: **Association of NOS1AP genetic variants with QT interval duration in families from the Diabetes Heart Study.** *Diabetes* 2008, **57:**1108-1114.

11. Tobin MD, Kahonen M, Braund P, Nieminen T, Hajat C, Tomaszewski M, Viik J, Lehtinen R, Ng GA, Macfarlane PW, et al: **Gender and effects of a common genetic variant in the NOS1 regulator NOS1AP on cardiac repolarization in 3761 individuals from two independent populations.** *Int J Epidemiol* 2008, **37:**1132-1141.

12. Sotoodehnia N, Li G, Johnson CO, Lemaitre RN, Rice KM, Rea TD, Siscovick DS: **Genetic variation in angiotensin-converting enzyme-related pathways associated with sudden cardiac arrest risk.** *Heart Rhythm* 2009, **6:**1306-1314.

13. Arking DE, Reinier K, Post W, Jui J, Hilton G, O'Connor A, Prineas RJ, Boerwinkle E, Psaty BM, Tomaselli GF, et al: **Genome-wide association study identifies GPC5 as a novel genetic locus protective against sudden cardiac arrest.** *PLoS One*, **5:**e9879.

14. Lehnart SE, Ackerman MJ, Benson DW, Jr., Brugada R, Clancy CE, Donahue JK, George AL, Jr., Grant AO, Groft SC, January CT, et al: **Inherited arrhythmias: a National Heart, Lung, and Blood Institute and Office of Rare Diseases workshop consensus report about the diagnosis, phenotyping, molecular mechanisms, and therapeutic approaches for primary cardiomyopathies of gene mutations affecting ion channel function.** *Circulation* 2007, **116:**2325-2345.

15. Conde L, Vaquerizas JM, Dopazo H, Arbiza L, Reumers J, Rousseau F, Schymkowitz J, Dopazo J: **PupaSuite: finding functional single nucleotide polymorphisms for large-scale genotyping purposes.** *Nucleic Acids Res* 2006, **34:**W621-625.

16. Ramensky V, Bork P, Sunyaev S: **Human non-synonymous SNPs: server and survey.** *Nucleic Acids Res* 2002, **30:**3894-3900.
