# Supplementary material for: GWAS for discovery and replication of genetic loci associated with sudden cardiac arrest in patients with coronary artery disease
Source: BMC Cardiovasc Disord. 2011 Jun 10;11:29. doi: 10.1186/1471-2261-11-29 (PMC3141757; doi:10.1186/1471-2261-11-29)
Supplement: Additional file 2 — Empirically-determined quality control thresholds for SNP Call Rate and Hardy-Weinberg Equilibrium. In Panel A, the region of the point of inflection in SNP call rate was rendered with call rate on the Y axis and SNPs ordered by call rate on the X-axis. In Panel B, HWE p-values are plotted along the Y-axis with SNPs ordered by HWE p-value on the X-axis, the inflection point (p < 0.00015) indicated. [file 1471-2261-11-29-S2.DOC]

### Additional file 7

**Title: Empirically-determined quality control thresholds for SNP Call Rate and Hardy-Weinberg Equilibrium**

# Description: In Panel A, the region of the point of inflection in SNP call rate was rendered with call rate on the Y axis and SNPs ordered by call rate on the X-axis. In Panel B, HWE p-values are plotted along the Y-axis with SNPs ordered by HWE p-value on the X-axis, the inflection point (p<0.00015) indicated.

### Suppl. Figure 1 - Empirically-determined quality control thresholds for SNP Call Rate and Hardy-Weinberg Equilibrium

**A) Genome-wide SNP Call Rate.**


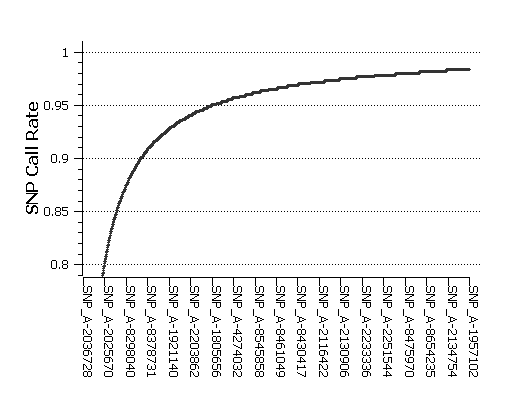


**B) Plot of HWE Chi Square (Fisher’s Exact).**

**
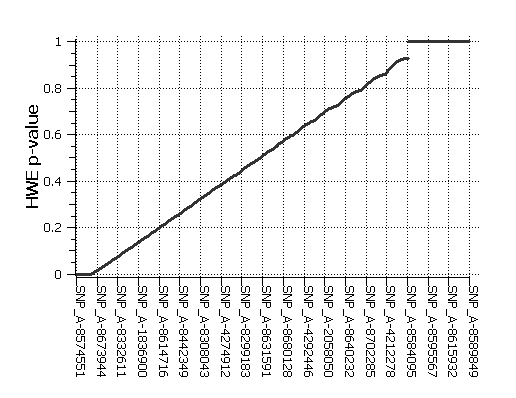
**

**p<0.00015**
