# Supplementary material for: GWAS for discovery and replication of genetic loci associated with sudden cardiac arrest in patients with coronary artery disease
Source: BMC Cardiovasc Disord. 2011 Jun 10;11:29. doi: 10.1186/1471-2261-11-29 (PMC3141757; doi:10.1186/1471-2261-11-29)

### Additional file 9

**Title: Association localization plots for ZNF385B verifying minimal overlap in tagSNP selection**

# Description: In Panel A, the region of the point of inflection in SNP call rate was rendered with call rate on the Y axis and SNPs ordered by call rate on the X-axis. In Panel B, HWE p-values are plotted along the Y-axis with SNPs ordered by HWE p-value on the X-axis, the inflection point (p<0.00015) indicated.


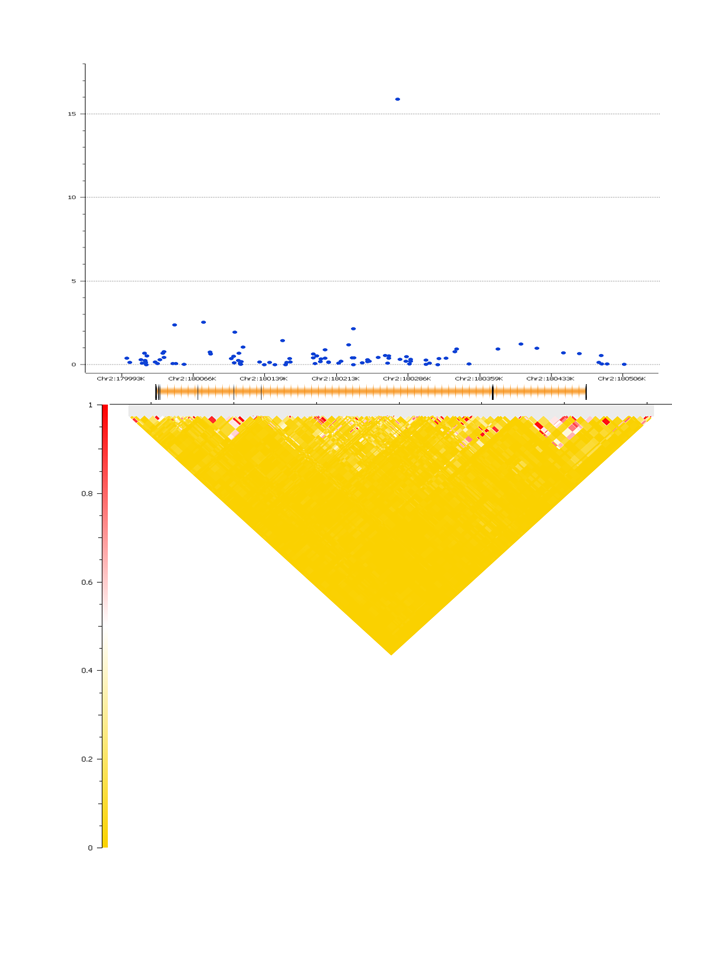

Supplement: Additional file 4 — Association localization plots for ZNF385B verifying minimal overlap in tagSNP selection. In Panel A, the region of the point of inflection in SNP call rate was rendered with call rate on the Y axis and SNPs ordered by call rate on the X-axis. In Panel B, HWE p-values are plotted along the Y-axis with SNPs ordered by HWE p-value on the X-axis, the inflection point (p < 0.00015) indicated. [file 1471-2261-11-29-S4.DOC]
