# Supplementary material for: GWAS for discovery and replication of genetic loci associated with sudden cardiac arrest in patients with coronary artery disease
Source: BMC Cardiovasc Disord. 2011 Jun 10;11:29. doi: 10.1186/1471-2261-11-29 (PMC3141757; doi:10.1186/1471-2261-11-29)

### Additional file 11

**Title: Association localization plots for ESR1 verifying minimal overlap in tagSNP selection**

# Description: Results for tagSNPs used in the discovery phase (adjusted for principal components) are presented as circles. Negative LOG p-values are provided on the Y axis. The X axis corresponds to the locations of SNPs. The p-value obtained with the most highly associated SNP is indicated with a green arrow. The LD relationship (R2) of the tagSNPs analyzed in the pilot GWAS are shown below the graph with shared variance between tagSNP pairs ranging from gold (low shared variance) to red (complete shared variance).


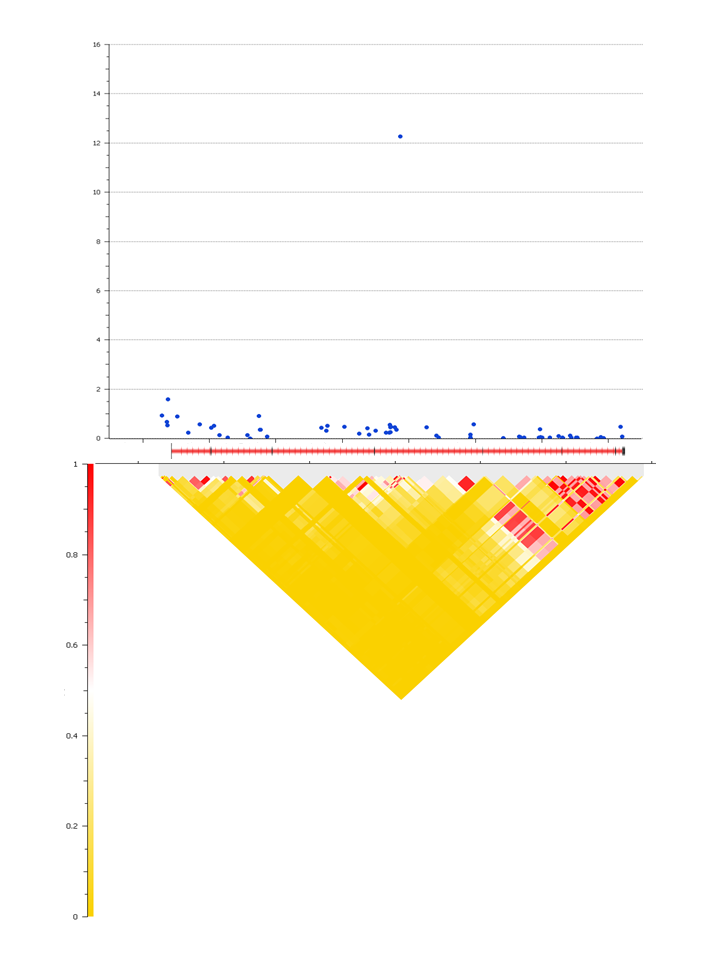

Supplement: Additional file 6 — Association localization plots for ESR1 verifying minimal overlap in tagSNP selection. Results for tagSNPs used in the discovery phase (adjusted for principal components) are presented as circles. Negative LOG p-values are provided on the Y axis. The × axis corresponds to the locations of SNPs. The p-value obtained with the most highly associated SNP is indicated with a green arrow. The LD relationship (R2) of the tagSNPs analyzed in the pilot GWAS are shown below the graph with shared variance between tagSNP pairs ranging from gold (low shared variance) to red (complete shared variance). [file 1471-2261-11-29-S6.DOC]
