# Supplementary material for: GWAS for discovery and replication of genetic loci associated with sudden cardiac arrest in patients with coronary artery disease
Source: BMC Cardiovasc Disord. 2011 Jun 10;11:29. doi: 10.1186/1471-2261-11-29 (PMC3141757; doi:10.1186/1471-2261-11-29)

### Additional file 3

**Title: GWAS Q-Q plots for the dominant model of component control groups**

# Description: Q-Q plots of the negative log10 P values for crude (Panels A, C, and E) and PCA-corrected (Panels B, D, and F) correlation tests for genome-wide association across the genome are shown for the Renal Transplant Donor Controls as compared to the Narcolepsy Controls (Panels A and D), HapMap Controls as compared to the Narcolepsy Controls (Panels B and D), and the HapMap Controls as compared to the Renal Transplant Donor Controls (Panels C and F).

A] D]


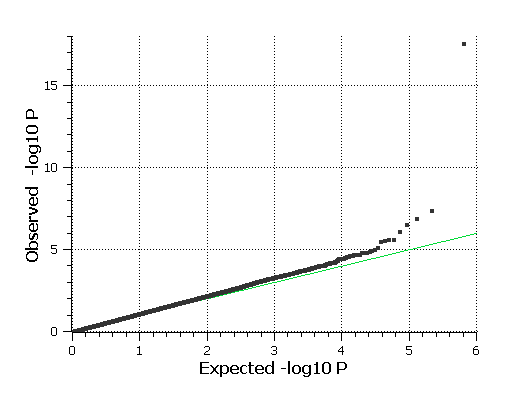

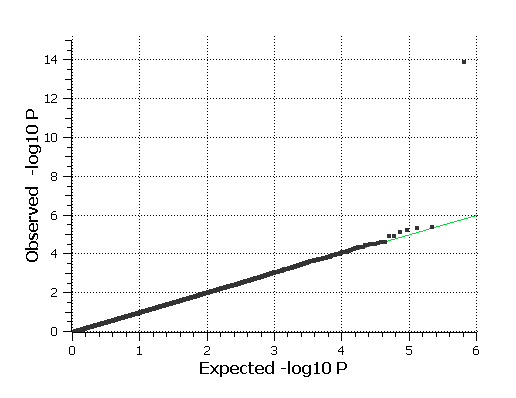


B] E]


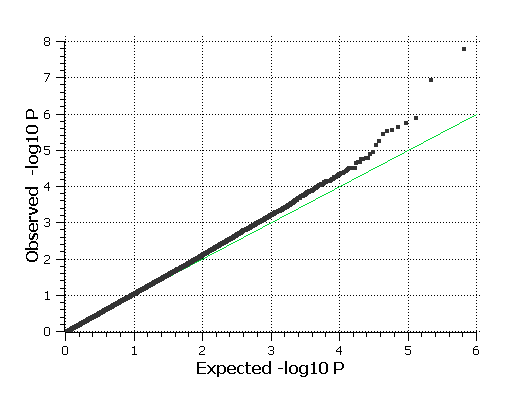

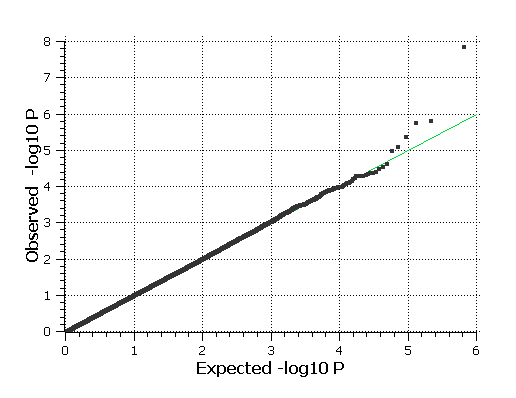


C] F]


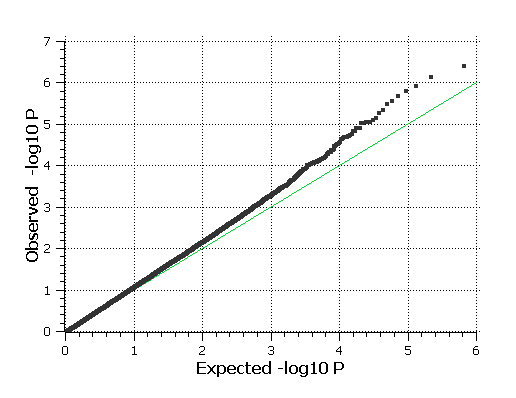

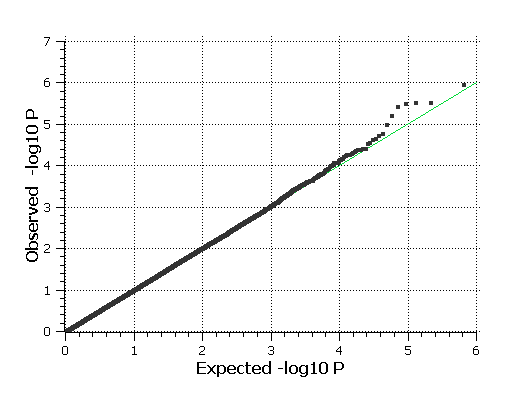

Supplement: Additional file 10 — GWAS Q-Q plots for the dominant model of component control groups. Q-Q plots of the negative log10 P values for crude (Panels A, C, and E) and PCA-corrected (Panels B, D, and F) correlation tests for genome-wide association across the genome are shown for the Renal Transplant Donor Controls as compared to the Narcolepsy Controls (Panels A and D), HapMap Controls as compared to the Narcolepsy Controls (Panels B and D), and the HapMap Controls as compared to the Renal Transplant Donor Controls (Panels C and F). [file 1471-2261-11-29-S10.DOC]
