# Supplementary material for: GWAS for discovery and replication of genetic loci associated with sudden cardiac arrest in patients with coronary artery disease
Source: BMC Cardiovasc Disord. 2011 Jun 10;11:29. doi: 10.1186/1471-2261-11-29 (PMC3141757; doi:10.1186/1471-2261-11-29)
Supplement: Additional file 12 — List of top 300 SNPs identified after correction for population stratification. SNPs also listed in Table 2 (significant after Bonferroni correction) are boxed for ease of identification. Genes with evidence of a role in cardiovascular phenotype are rendered in italics. Genes that are mechanistically recognized candidates for SCA are rendered in italicized bold font. Unless otherwise indicated, a recessive genetic model fit the data best. Abbreviations: rsID, Reference Sequence IDentifier for a given single nucleotide polymorphism (SNP); PPCA, p-value for the PCA-corrected test statistic (i.e., Armitage Trend Test for the Additive model, Fisher's Exact Test for Dominant and Recessive Models); PUNADJ, p-value for the unadjusted test statistic (i.e., Correlation Test); ORPCA-ADJ (95% CI), the odds ratio and 95% confidence interval for the test statistic; PPCA-ADJ, p-value for the test statistic adjusted for PCA, age, and sex; MAF, minor allele frequency; PHWE, p-value for the Hardy-Weinberg test statistic; Region; chromosome and band position for the variation; Position, nucleotide position for the variation. [file 1471-2261-11-29-S12.DOC]

### Additional file 5: Supplementary Table 1

**Title: List of top 300 SNPs identified after correction for population stratification**

### Description: SNPs also listed in Table 2 (significant after Bonferroni correction) are boxed for ease of identification. Genes with evidence of a role in cardiovascular phenotype are rendered in italics. Genes that are mechanistically recognized candidates for SCA are rendered in italicized bold font. Unless otherwise indicated, a recessive genetic model fit the data best. Abbreviations: rsID, Reference Sequence IDentifier for a given single nucleotide polymorphism (SNP); PPCA, p-value for the PCA-corrected test statistic (i.e., Armitage Trend Test for the Additive model, Fisher’s Exact Test for Dominant and Recessive Models ); PUNADJ, p-value for the unadjusted test statistic (i.e., Correlation Test); ORPCA-ADJ (95% CI), the odds ratio and 95% confidence interval for the test statistic; PPCA-ADJ, p-value for the test statistic adjusted for PCA, age, and sex; MAF, minor allele frequency; PHWE, p-value for the Hardy-Weinberg test statistic; Region; chromosome and band position for the variation; Position, nucleotide position for the variation.

| **Gene** | **rs ID** | **P_PCA_** | **P_UNADJ_** | **OR_ADJ_ (95% CI)** | **P_ADJ_** | **MAF** | **Call Rate** | **P_HWE_** | **Region** | **Position** |
| --- | --- | --- | --- | --- | --- | --- | --- | --- | --- | --- |
| **a) Additive** |  |  |  |  |  |  |  |  |  |  |
| SPSB1 | rs478103 | 1.44E-05 | 1.62E-05 | 1.15 (1.078, 1.224) | 2.02E-05 | 0.079 | 0.975 | 0.154982 | 1p36.22 | 9324990 |
|  | rs4584347 | 4.97E-04 | 8.73E-03 | 0.94 (0.912, 0.974) | 4.66E-04 | 0.443 | 0.995 | 0.859093 | 1p31.1 | 73914426 |
| SPTA1 | rs703115 | 4.98E-04 | 6.10E-04 | 1.06 (1.027, 1.098) | 4.33E-04 | 0.422 | 1.000 | 0.315806 | 1q23.1 | 156909661 |
| OR6K6 | rs167821 | 2.90E-05 | 2.75E-04 | 0.93 (0.9, 0.966) | 1.07E-04 | 0.319 | 0.995 | 0.694869 | 1q23.1 | 156968813 |
| OR6K6 | rs2564856 | 2.05E-04 | 1.58E-03 | 0.94 (0.911, 0.974) | 4.78E-04 | 0.435 | 0.995 | 0.423926 | 1q23.1 | 156979251 |
|  | rs1018391 | 7.18E-05 | 3.97E-04 | 0.93 (0.901, 0.962) | 2.16E-05 | 0.469 | 1.000 | 0.929963 | 1q24.3 | 169607054 |
|  | rs1044925 | 2.19E-05 | 1.20E-05 | 1.07 (1.032, 1.107) | 2.26E-04 | 0.351 | 0.995 | 0.192536 | 1q25.2 | 177590361 |
|  | rs4268383 | 8.42E-05 | 1.51E-04 | 1.13 (1.06, 1.215) | 3.17E-04 | 0.057 | 1.000 | 0.089102 | 1q44 | 246205143 |
|  | rs7601231 | 2.83E-04 | 1.48E-03 | 1.07 (1.031, 1.113) | 4.30E-04 | 0.226 | 0.992 | 0.061802 | 2p25.3 | 4154567 |
| *ROCK2* | *rs12479227* | *3.92E-05* | *7.22E-05* | *1.17 (1.086, 1.255)* | *3.03E-05* | *0.054* | *0.985* | *1.000000* | *2p25.1* | *11311804* |
|  | rs917732 | 1.87E-04 | 1.04E-03 | 1.07 (1.036, 1.107) | 4.81E-05 | 0.409 | 0.997 | 0.579165 | 2p22.2 | 37685057 |
| TET3 | rs12991192 | 2.97E-04 | 1.95E-03 | 1.08 (1.035, 1.117) | 1.86E-04 | 0.223 | 1.000 | 0.139172 | 2p13.1 | 74163174 |
|  | rs7597912 | 4.05E-05 | 2.26E-04 | 1.09 (1.045, 1.13) | 3.17E-05 | 0.250 | 0.975 | 0.380465 | 2p12 | 79631620 |
| *CXCR4* | *rs16835520* | *5.73E-04* | *7.32E-05* | *1.15 (1.068, 1.228)* | *1.54E-04* | *0.055* | *0.992* | *0.251349* | *2q22.1* | *137032611* |
| LOC285141 | rs3764916 | 2.17E-04 | 4.71E-04 | 1.07 (1.031, 1.106) | 2.92E-04 | 0.329 | 0.998 | 0.536774 | 2q31.1 | 171357648 |
| GAD1 | rs6736129 | 1.15E-04 | 5.68E-05 | 1.08 (1.038, 1.123) | 1.37E-04 | 0.242 | 0.990 | 0.521380 | 2q31.1 | 171370585 |
|  | rs4668374 | 8.18E-05 | 9.01E-04 | 0.93 (0.887, 0.966) | 3.80E-04 | 0.188 | 0.995 | 0.588753 | 2q31.1 | 171777737 |
| IKZF2 | rs935092 | 9.14E-05 | 4.48E-04 | 1.14 (1.073, 1.216) | 3.39E-05 | 0.074 | 0.998 | 0.711551 | 2q34 | 213729570 |
|  | rs12634491 | 3.41E-04 | 2.18E-03 | 1.14 (1.066, 1.221) | 1.44E-04 | 0.063 | 0.998 | 1.000000 | 3p14.1 | 70412228 |
|  | rs2916438 | 1.15E-04 | 2.47E-04 | 1.08 (1.037, 1.124) | 1.92E-04 | 0.223 | 1.000 | 0.503011 | 4p16.2 | 4336766 |
|  | rs10026526 | 3.77E-05 | 1.06E-04 | 0.94 (0.907, 0.968) | 8.84E-05 | 0.477 | 0.970 | 0.378038 | 4p15.2 | 24065030 |
| ZCCHC4 | rs6826966 | 4.23E-04 | 1.35E-04 | 1.12 (1.059, 1.192) | 1.26E-04 | 0.096 | 0.980 | 0.038186 | 4p15.2 | 24917441 |
|  | rs6419096 | 5.69E-05 | 1.55E-04 | 1.09 (1.049, 1.14) | 3.32E-05 | 0.195 | 0.992 | 0.649489 | 4p14 | 36657202 |
|  | rs13141189 | 3.00E-05 | 4.59E-06 | 1.08 (1.038, 1.131) | 2.40E-04 | 0.179 | 0.992 | 0.505403 | 4q28.3 | 131340842 |
|  | rs4516871 | 5.31E-04 | 6.95E-04 | 1.09 (1.042, 1.135) | 1.24E-04 | 0.176 | 0.997 | 0.746144 | 5p13.3 | 31213385 |
|  | rs282072 | 4.28E-04 | 2.08E-04 | 0.94 (0.909, 0.971) | 2.22E-04 | 0.422 | 1.000 | 0.858684 | 5q21.1 | 100006537 |
| LOC441108 | rs17715481 | 3.89E-05 | 3.06E-04 | 1.15 (1.081, 1.233) | 2.17E-05 | 0.068 | 0.970 | 1.000000 | 5q31.1 | 131843283 |
|  | rs262023 | 1.85E-04 | 1.39E-05 | 0.94 (0.906, 0.967) | 9.24E-05 | 0.466 | 0.998 | 0.660430 | 5q35.3 | 177957693 |
| CDKN1A | rs876581 | 2.30E-04 | 2.15E-05 | 1.14 (1.067, 1.212) | 7.89E-05 | 0.070 | 1.000 | 0.225307 | 6p21.31 | 36763423 |
|  | rs190759 | 3.58E-06 | 1.07E-07 | 1.11 (1.061, 1.158) | 4.51E-06 | 0.176 | 1.000 | 0.232188 | 6p12.3 | 51095727 |
|  | rs12211338 | 6.53E-05 | 3.20E-06 | 1.13 (1.067, 1.197) | 3.08E-05 | 0.090 | 1.000 | 0.345024 | 6p12.2 | 51161681 |
| *FYN* | *rs2815787* | *2.51E-04* | *4.33E-05* | *1.08 (1.04, 1.131)* | *1.69E-04* | *0.187* | *1.000* | *0.752994* | *6q21* | *112439086* |
| C6orf118 | rs11751409 | 4.26E-04 | 6.95E-03 | 1.07 (1.035, 1.108) | 8.78E-05 | 0.358 | 1.000 | 0.557687 | 6q26 | 164361831 |
| C6orf118 | rs2146031 | 8.93E-05 | 3.00E-05 | 1.16 (1.086, 1.243) | 1.40E-05 | 0.061 | 1.000 | 0.353474 | 6q27 | 165345328 |
| *PIK3CG* | *rs342307* | *4.65E-04* | *4.53E-03* | *0.94 (0.908, 0.971)* | *2.02E-04* | *0.455* | *0.993* | *0.377105* | *7q22.3* | *106169554* |
| ***NOS3*** | ***rs17173656*** | ***5.41E-05*** | ***3.09E-04*** | ***1.13 (1.068, 1.19)*** | ***1.53E-05*** | ***0.102*** | ***1.000*** | ***0.786064*** | ***7q36.1*** | ***150229592*** |
| ***NOS3*** | ***rs17173658*** | ***6.24E-05*** | ***6.87E-05*** | ***1.15 (1.07, 1.229)*** | ***1.21E-04*** | ***0.059*** | ***0.998*** | ***0.317805*** | ***7q36.1*** | ***150242094*** |
|  | rs16921991 | 3.09E-04 | 1.09E-04 | 0.93 (0.895, 0.969) | 4.67E-04 | 0.216 | 0.998 | 1.000000 | 8q12.1 | 58386566 |
| T1560 | rs1317890 | 4.76E-04 | 5.90E-04 | 0.93 (0.901, 0.968) | 2.28E-04 | 0.286 | 1.000 | 0.603805 | 8q12.1 | 58452664 |
| T1560 | rs7845730 | 2.18E-04 | 3.84E-04 | 0.92 (0.89, 0.961) | 6.99E-05 | 0.255 | 0.997 | 0.075395 | 8q12.1 | 58512321 |
| *LAPTM4B* | *rs1136594* | *2.26E-04* | *1.55E-05* | *0.94 (0.903, 0.97)* | *2.61E-04* | *0.360* | *0.997* | *0.113710* | *8q22.1* | *98933052* |
| BICC1 | rs3910871 | 5.16E-04 | 1.01E-02 | 1.12 (1.052, 1.195) | 4.48E-04 | 0.084 | 0.955 | 0.097832 | 10q21.1 | 60327507 |
|  | rs2207658 | 1.21E-04 | 1.19E-04 | 1.1 (1.045, 1.167) | 4.22E-04 | 0.103 | 0.980 | 0.578934 | 10q23.1 | 83592583 |
|  | rs12358914 | 1.70E-05 | 6.10E-05 | 1.08 (1.039, 1.123) | 1.18E-04 | 0.215 | 0.995 | 0.209663 | 10q25.2 | 112415168 |
| PLEKHG7 | rs10219495 | 4.53E-06 | 3.51E-05 | 1.14 (1.078, 1.206) | 4.73E-06 | 0.104 | 0.964 | 0.409236 | 12q22 | 91414651 |
|  | rs1823172 | 5.72E-09 | 4.03E-11 | 1.17 (1.102, 1.245) | 5.22E-07 | 0.076 | 1.000 | 1.000000 | 12q24.32 | 126358567 |
| SLC46A3 | rs327118 | 4.52E-04 | 6.87E-04 | 0.94 (0.906, 0.967) | 8.48E-05 | 0.458 | 0.997 | 1.000000 | 13q12.3 | 28404175 |
|  | rs9579477 | 1.12E-04 | 1.01E-04 | 1.08 (1.043, 1.12) | 2.33E-05 | 0.323 | 0.992 | 0.401101 | 13q12.3 | 29206054 |
|  | rs9603692 | 1.04E-04 | 1.22E-05 | 1.06 (1.028, 1.099) | 3.75E-04 | 0.476 | 0.997 | 0.328406 | 13q14.11 | 39636316 |
| MYO16 | rs9587725 | 3.56E-05 | 9.70E-07 | 1.16 (1.076, 1.253) | 1.33E-04 | 0.051 | 0.998 | 0.525757 | 13q33.3 | 108389041 |
| LRFN5 | rs17212596 | 2.75E-04 | 1.39E-03 | 1.07 (1.032, 1.112) | 3.52E-04 | 0.263 | 0.980 | 1.000000 | 14q21.3 | 43364104 |
|  | rs4886707 | 1.13E-04 | 6.14E-07 | 1.09 (1.044, 1.128) | 3.51E-05 | 0.255 | 0.978 | 0.614329 | 15q24.2 | 73542520 |
| CHRNA5 | rs481134 | 2.01E-04 | 3.41E-05 | 0.94 (0.91, 0.97) | 1.55E-04 | 0.397 | 1.000 | 0.071459 | 15q25.1 | 76664618 |
| CHRNB4 | rs950776 | 9.16E-07 | 2.74E-07 | 0.92 (0.886, 0.947) | 3.55E-07 | 0.318 | 0.983 | 0.097477 | 15q25.1 | 76713073 |
|  | rs11637890 | 4.56E-04 | 2.09E-04 | 0.94 (0.908, 0.971) | 2.25E-04 | 0.354 | 0.983 | 0.156730 | 15q25.1 | 76722474 |
|  | rs4337270 | 5.88E-04 | 2.01E-03 | 1.1 (1.046, 1.147) | 1.19E-04 | 0.135 | 0.990 | 0.153277 | 15q25.3 | 84343916 |
|  | rs11654550 | 4.37E-04 | 2.79E-03 | 1.06 (1.028, 1.101) | 4.68E-04 | 0.445 | 0.992 | 0.087057 | 17p12 | 13499770 |
|  | rs2251393 | 6.00E-07 | 1.79E-07 | 1.13 (1.076, 1.179) | 3.74E-07 | 0.146 | 1.000 | 0.545373 | 17q23.2 | 58132664 |
|  | rs12451315 | 1.53E-05 | 8.33E-05 | 1.07 (1.035, 1.108) | 8.84E-05 | 0.474 | 0.969 | 0.367163 | 17q25.2 | 72883721 |
| C18orf1 | rs8099604 | 3.20E-04 | 2.16E-04 | 0.91 (0.866, 0.948) | 1.97E-05 | 0.175 | 1.000 | 0.031545 | 18p11.21 | 13390350 |
| C18orf1 | rs9807144 | 2.56E-04 | 1.14E-04 | 0.92 (0.881, 0.958) | 8.51E-05 | 0.223 | 0.997 | 0.039883 | 18p11.21 | 13391662 |
| NETO1 | rs1943829 | 6.37E-05 | 2.67E-05 | 1.1 (1.047, 1.154) | 1.63E-04 | 0.131 | 1.000 | 1.000000 | 18q22.3 | 69107759 |
|  | rs11083866 | 4.36E-04 | 2.29E-03 | 1.07 (1.031, 1.111) | 3.64E-04 | 0.249 | 0.988 | 0.456920 | 19q12 | 34428182 |
| PAK7 | rs6140980 | 1.52E-04 | 3.40E-05 | 1.14 (1.064, 1.226) | 2.55E-04 | 0.059 | 0.995 | 0.620371 | 20p12.2 | 9533479 |
| CDH4 | rs944260 | 6.32E-07 | 2.17E-07 | 1.1 (1.058, 1.144) | 1.84E-06 | 0.240 | 0.998 | 0.152093 | 20q13.33 | 59676806 |
|  | rs2835099 | 3.94E-04 | 9.95E-05 | 1.18 (1.088, 1.272) | 4.73E-05 | 0.051 | 0.965 | 1.000000 | 21q22.12 | 35994540 |
| **b) Dominant** |  |  |  |  |  |  |  |  |  |  |
| TMCO4 | rs3767216 | 2.32E-05 | 1.63E-04 | 0.9 (0.86, 0.946) | 2.77E-05 | 0.337 | 0.987 | 0.288262 | 1p36.13 | 20012927 |
| *FMO1* | *rs6660565* | *1.66E-05* | *1.11E-04* | *0.89 (0.849, 0.933)* | *1.54E-06* | *0.283* | *0.992* | *0.833835* | *1q24.3* | *169527817* |
|  | rs12082991 | 3.78E-04 | 8.06E-04 | 1.14 (1.068, 1.226) | 1.46E-04 | 0.070 | 0.993 | 0.410505 | 1q42.2 | 231334710 |
|  | rs10925917 | 7.26E-05 | 3.17E-04 | 1.1 (1.051, 1.157) | 7.88E-05 | 0.378 | 0.993 | 0.500130 | 1q43 | 237783742 |
|  | rs6756474 | 4.93E-05 | 1.43E-04 | 1.09 (1.043, 1.147) | 2.41E-04 | 0.299 | 0.987 | 0.660819 | 2p13.1 | 74136214 |
| REG3A | rs10206130 | 3.40E-04 | 4.58E-04 | 1.1 (1.053, 1.158) | 4.13E-05 | 0.302 | 1.000 | 0.450005 | 2p12 | 79604099 |
| *RAB3GAP1* | *rs7603310* | *2.99E-05* | *1.37E-05* | *1.15 (1.064, 1.234)* | *3.40E-04* | *0.060* | *0.988* | *0.320367* | *2q21.3* | *135618690* |
| ARHGAP15 | rs1257351 | 5.44E-06 | 2.14E-05 | 1.14 (1.067, 1.219) | 1.20E-04 | 0.079 | 0.997 | 0.145654 | 2q22.2 | 144347638 |
| *ZEB2* | *rs16823732* | *8.51E-05* | *5.78E-05* | *1.15 (1.071, 1.237)* | *1.44E-04* | *0.066* | *0.997* | *0.155156* | *2q22.3* | *144921228* |
|  | rs10177533 | 7.31E-05 | 6.41E-05 | 1.1 (1.053, 1.158) | 4.65E-05 | 0.234 | 1.000 | 0.120172 | 2q22.3 | 148200533 |
| ORC4L | rs17693310 | 3.35E-05 | 8.81E-06 | 1.11 (1.054, 1.162) | 5.86E-05 | 0.192 | 0.998 | 0.876659 | 2q23.1 | 148464448 |
|  | rs10183640 | 3.40E-06 | 7.59E-06 | 1.12 (1.075, 1.176) | 5.04E-07 | 0.349 | 0.954 | 0.058465 | 2q24.1 | 158488450 |
| GAD1 | rs10191129 | 2.12E-04 | 6.98E-04 | 1.1 (1.051, 1.154) | 6.19E-05 | 0.267 | 1.000 | 0.556692 | 2q31.1 | 171396839 |
| IRS1 | rs13431179 | 1.11E-04 | 4.15E-05 | 1.13 (1.057, 1.212) | 4.29E-04 | 0.075 | 0.995 | 0.123946 | 2q36.3 | 227318485 |
| C3orf67 | rs2366846 | 2.18E-05 | 1.71E-05 | 0.91 (0.866, 0.96) | 4.53E-04 | 0.175 | 0.990 | 0.887130 | 3p14.2 | 59680934 |
|  | rs696375 | 4.26E-04 | 3.53E-04 | 1.1 (1.054, 1.158) | 3.89E-05 | 0.304 | 0.972 | 1.000000 | 3q13.12 | 109214773 |
| *FSTL1* | *rs1259337* | *7.05E-05* | *1.21E-04* | *1.1 (1.051, 1.155)* | *6.86E-05* | *0.279* | *0.998* | *0.112080* | *3q13.33* | *121648084* |
| EPHB1 | rs7627183 | 6.72E-05 | 4.68E-04 | 1.09 (1.04, 1.144) | 3.76E-04 | 0.323 | 0.998 | 0.754942 | 3q22.2 | 136325930 |
| CLSTN2 | rs11708189 | 2.07E-05 | 7.97E-05 | 0.89 (0.849, 0.933) | 1.74E-06 | 0.345 | 0.977 | 0.701039 | 3q23 | 141658050 |
| PLSCR4 | rs17349248 | 4.87E-04 | 1.25E-03 | 1.12 (1.062, 1.179) | 2.96E-05 | 0.153 | 0.988 | 0.708975 | 3q24 | 147408262 |
|  | rs340229 | 3.97E-04 | 1.53E-04 | 0.91 (0.859, 0.957) | 4.41E-04 | 0.496 | 0.995 | 0.724161 | 3q25.33 | 160110339 |
|  | rs1488895 | 2.85E-05 | 1.41E-04 | 1.12 (1.055, 1.191) | 2.55E-04 | 0.099 | 1.000 | 0.087436 | 3q26.32 | 178060524 |
| KCNIP4 | rs13110425 | 1.78E-04 | 7.18E-04 | 1.1 (1.053, 1.157) | 4.59E-05 | 0.297 | 1.000 | 0.587167 | 4p15.31 | 20960603 |
| IGFBP7 | rs2412775 | 6.43E-05 | 4.63E-05 | 1.1 (1.043, 1.157) | 4.15E-04 | 0.452 | 0.995 | 0.323102 | 4q12 | 57614946 |
|  | rs6532316 | 3.76E-04 | 7.95E-04 | 0.9 (0.851, 0.952) | 2.56E-04 | 0.122 | 1.000 | 1.000000 | 4q21.1 | 77417780 |
|  | rs17068150 | 3.04E-04 | 1.89E-03 | 1.12 (1.055, 1.182) | 1.59E-04 | 0.117 | 0.998 | 0.489437 | 4q34.3 | 180922219 |
|  | rs6866502 | 1.76E-05 | 3.82E-04 | 0.92 (0.874, 0.962) | 4.53E-04 | 0.238 | 1.000 | 0.907755 | 5p14.3 | 23255456 |
| PART1 | rs10939851 | 4.02E-04 | 3.51E-03 | 1.12 (1.06, 1.174) | 3.43E-05 | 0.160 | 1.000 | 0.729965 | 5q12.1 | 59877118 |
|  | rs2148975 | 1.59E-04 | 1.98E-05 | 0.9 (0.86, 0.947) | 3.14E-05 | 0.208 | 0.959 | 0.443373 | 6p25.1 | 6883927 |
| TXNDC5 | rs2748364 | 2.71E-04 | 1.50E-03 | 0.9 (0.851, 0.947) | 8.15E-05 | 0.488 | 1.000 | 0.929892 | 6p24.3 | 7917476 |
|  | rs2734971 | 2.17E-05 | 1.58E-04 | 1.13 (1.067, 1.189) | 1.92E-05 | 0.500 | 0.959 | 0.791309 | 6p21.33 | 29942428 |
|  | rs17053610 | 9.63E-05 | 2.38E-04 | 1.13 (1.061, 1.201) | 1.46E-04 | 0.085 | 0.967 | 0.324051 | 6q16.2 | 99872505 |
| PREP | rs9399956 | 4.28E-07 | 2.46E-06 | 1.1 (1.053, 1.15) | 2.68E-05 | 0.263 | 0.952 | 0.043037 | 6q21 | 106443056 |
|  | rs7830263 | 3.90E-04 | 2.73E-04 | 1.1 (1.043, 1.159) | 4.15E-04 | 0.151 | 1.000 | 0.850591 | 8p23.2 | 4611638 |
|  | rs12164204 | 1.65E-05 | 2.88E-06 | 1.1 (1.057, 1.154) | 1.16E-05 | 0.376 | 0.950 | 0.016600 | 8p23.2 | 5953489 |
| EFCAB1 | rs12155623 | 1.28E-06 | 2.68E-06 | 0.88 (0.833, 0.921) | 2.73E-07 | 0.425 | 0.977 | 0.368154 | 8q11.21 | 49974754 |
| SNTG1 | rs16915013 | 6.69E-05 | 1.04E-05 | 1.12 (1.051, 1.184) | 3.64E-04 | 0.102 | 0.995 | 0.159610 | 8q11.22 | 51623563 |
|  | rs10809903 | 3.15E-04 | 5.90E-04 | 0.91 (0.866, 0.958) | 2.71E-04 | 0.185 | 0.988 | 0.166608 | 9p24.3 | 1309470 |
| *PCSK5* | *rs2842494* | *2.20E-04* | *3.93E-04* | *1.1 (1.046, 1.151)* | *1.51E-04* | *0.241* | *1.000* | *0.526103* | *9q21.13* | *78149229* |
|  | rs12342274 | 5.68E-05 | 4.02E-04 | 1.12 (1.056, 1.187) | 1.80E-04 | 0.105 | 0.997 | 0.428610 | 9q22.32 | 96320159 |
|  | rs7864831 | 9.28E-07 | 9.15E-06 | 1.17 (1.087, 1.25) | 1.86E-05 | 0.069 | 0.990 | 0.200084 | 9q22.32 | 96444415 |
| INSC | rs11023771 | 1.57E-04 | 4.53E-04 | 0.92 (0.874, 0.961) | 3.27E-04 | 0.281 | 0.998 | 0.753101 | 11p15.2 | 15878737 |
|  | rs11602432 | 1.68E-05 | 5.12E-06 | 0.91 (0.867, 0.958) | 2.49E-04 | 0.340 | 0.965 | 0.032602 | 11p14.3 | 25175721 |
| *WT1* | *rs5030316* | *1.06E-04* | *2.74E-05* | *1.13 (1.056, 1.201)* | *3.18E-04* | *0.088* | *1.000* | *0.047569* | *11p13* | *32366956* |
|  | rs12799766 | 3.24E-04 | 1.17E-03 | 1.09 (1.041, 1.148) | 3.74E-04 | 0.214 | 1.000 | 0.579999 | 11q23.3 | 116063637 |
| RASSF8 | rs1488827 | 2.30E-04 | 2.60E-04 | 1.12 (1.056, 1.184) | 1.37E-04 | 0.120 | 0.998 | 0.817307 | 12p12.1 | 25934686 |
| *RAPGEF3* | *rs11168228* | *2.18E-04* | *1.81E-04* | *1.09 (1.042, 1.146)* | *2.63E-04* | *0.309* | *0.997* | *0.004384* | *12q13.11* | *46437218* |
| *DCN* | *rs10777317* | *7.40E-05* | *9.82E-05* | *0.89 (0.849, 0.936)* | *4.89E-06* | *0.404* | *0.995* | *0.856710* | *12q21.33* | *90504505* |
| PLXNC1 | rs10777572 | 8.73E-05 | 2.09E-04 | 1.13 (1.066, 1.192) | 2.73E-05 | 0.114 | 0.974 | 0.339481 | 12q22 | 92977940 |
|  | rs10777845 | 4.59E-05 | 1.55E-04 | 0.89 (0.854, 0.938) | 4.61E-06 | 0.331 | 0.998 | 0.099602 | 12q23.1 | 96213899 |
| NBEA | rs1538005 | 4.05E-04 | 1.02E-03 | 1.09 (1.04, 1.144) | 3.55E-04 | 0.251 | 1.000 | 0.145283 | 13q13.2 | 33658437 |
| *LECT1* | *rs235777* | *5.50E-05* | *3.56E-05* | *0.91 (0.872, 0.958)* | *2.00E-04* | *0.319* | *0.997* | *0.556197* | *13q21.1* | *52238134* |
|  | rs7325927 | 5.66E-05 | 1.91E-05 | 1.11 (1.054, 1.16) | 4.61E-05 | 0.260 | 0.992 | 0.180752 | 13q33.3 | 107273514 |
|  | rs8010877 | 3.19E-06 | 2.21E-07 | 1.11 (1.056, 1.173) | 7.78E-05 | 0.469 | 0.980 | 0.534495 | 14q22.1 | 49910857 |
| TTC8 | rs1815281 | 2.84E-05 | 9.08E-05 | 1.1 (1.046, 1.158) | 2.43E-04 | 0.451 | 0.972 | 0.588762 | 14q31.3 | 88647166 |
| *DEGS2* | *rs7157599* | *9.14E-09* | *6.98E-11* | *1.13 (1.082, 1.185)* | *1.30E-07* | *0.312* | *0.952* | *0.830456* | *14q32.2* | *99695655* |
| BUB1B | rs12915249 | 2.96E-04 | 4.32E-04 | 1.09 (1.039, 1.143) | 4.02E-04 | 0.300 | 0.993 | 0.017044 | 15q15.1 | 38278003 |
| RHCG | rs7184034 | 5.42E-06 | 1.18E-05 | 1.1 (1.048, 1.153) | 1.21E-04 | 0.339 | 0.997 | 0.685731 | 15q26.1 | 87756165 |
| *DYNLRB2* | *rs6564756* | *4.24E-04* | *2.62E-04* | *1.09 (1.041, 1.147)* | *3.63E-04* | *0.336* | *0.974* | *0.353964* | *16q23.2* | *78949659* |
| BANP | rs12597574 | 1.24E-04 | 4.96E-04 | 1.09 (1.04, 1.144) | 3.98E-04 | 0.298 | 0.995 | 0.383791 | 16q24.2 | 86913928 |
| *PRKCA* | *rs11656279* | *4.38E-04* | *4.39E-03* | *1.16 (1.088, 1.245)* | *1.12E-05* | *0.075* | *1.000* | *0.476262* | *17q24.2* | *61968926* |
| TMEM162 | rs10422195 | 4.55E-04 | 1.57E-03 | 0.92 (0.874, 0.961) | 3.11E-04 | 0.252 | 1.000 | 0.367119 | 19q13.12 | 40415864 |
| NLRP11 | rs10419435 | 2.96E-04 | 3.65E-04 | 1.15 (1.071, 1.237) | 1.33E-04 | 0.067 | 1.000 | 0.195314 | 19q13.42 | 61003957 |
|  | rs6079058 | 3.24E-04 | 1.03E-03 | 1.1 (1.05, 1.154) | 7.63E-05 | 0.338 | 1.000 | 0.546667 | 20p12.1 | 13349065 |
|  | rs5992986 | 1.20E-05 | 1.51E-06 | 0.91 (0.868, 0.959) | 3.37E-04 | 0.386 | 0.990 | 0.022447 | 22q11.21 | 16925811 |
| SDF2L1 | rs861844 | 5.29E-06 | 1.80E-04 | 1.12 (1.059, 1.183) | 6.43E-05 | 0.144 | 0.977 | 0.073731 | 22q11.21 | 20336219 |
| **c) Recessive** |  |  |  |  |  |  |  |  |  |  |
| *PRDM16* | *rs2493285* | *3.60E-05* | *2.87E-03* | *1.87 (1.323, 2.632)* | *4.01E-04* | *0.086* | *0.992* | *0.064883* | *1p36.32* | *3323455* |
| CD48 | rs10489639 | 7.26E-05 | 1.99E-04 | 1.15 (1.07, 1.226) | 9.33E-05 | 0.372 | 0.993 | 0.846438 | 1q23.3 | 158946151 |
|  | rs12139648 | 1.05E-05 | 9.25E-04 | 1.57 (1.257, 1.972) | 8.38E-05 | 0.075 | 1.000 | 1.000000 | 1q24.1 | 164270995 |
| NMNAT2 | rs2078087 | 5.16E-05 | 9.32E-04 | 1.55 (1.214, 1.981) | 4.66E-04 | 0.104 | 0.998 | 0.208998 | 1q25.3 | 181625028 |
|  | rs10754677 | 3.38E-05 | 1.76E-04 | 1.12 (1.05, 1.189) | 4.62E-04 | 0.443 | 0.978 | 0.204420 | 1q43 | 237899723 |
| RSAD2 | rs4669114 | 3.32E-06 | 3.91E-04 | 1.54 (1.261, 1.868) | 2.11E-05 | 0.097 | 0.975 | 0.605701 | 2p25.2 | 6954072 |
| *E2F6* | *rs6716724* | *3.90E-08* | *7.56E-06* | *1.39 (1.22, 1.574)* | *6.38E-07* | *0.177* | *0.987* | *0.502756* | *2p25.1* | *11428399* |
| KLHL29 | rs17726419 | 3.18E-06 | 1.62E-05 | 1.17 (1.089, 1.251) | 1.47E-05 | 0.376 | 0.997 | 0.179435 | 2p24.1 | 23373617 |
|  | rs3820937 | 2.97E-05 | 2.90E-03 | 2.1 (1.493, 2.957) | 2.27E-05 | 0.078 | 1.000 | 0.102697 | 2p23.3 | 26470128 |
| ACYP2 | rs1559040 | 1.04E-09 | 2.11E-06 | 1.54 (1.32, 1.787) | 3.76E-08 | 0.111 | 0.990 | 1.000000 | 2p16.2 | 54201254 |
|  | rs13020375 | 1.30E-05 | 6.59E-05 | 1.13 (1.058, 1.212) | 3.81E-04 | 0.370 | 1.000 | 1.000000 | 2p14 | 64682655 |
|  | rs2108482 | 2.51E-05 | 1.14E-04 | 1.16 (1.081, 1.238) | 2.92E-05 | 0.387 | 0.993 | 0.217742 | 2p13.3 | 70678969 |
|  | rs4851109 | 4.36E-05 | 9.39E-04 | 1.61 (1.279, 2.025) | 5.45E-05 | 0.086 | 0.997 | 0.757505 | 2q12.2 | 105984047 |
| DARS | rs309161 | 7.24E-05 | 5.41E-04 | 1.26 (1.107, 1.425) | 4.16E-04 | 0.174 | 1.000 | 0.868521 | 2q21.3 | 136405219 |
|  | rs707040 | 1.18E-05 | 6.89E-05 | 1.17 (1.096, 1.259) | 6.46E-06 | 0.379 | 0.993 | 0.246839 | 2q24.1 | 154934923 |
|  | rs13022357 | 2.39E-08 | 2.97E-05 | 1.72 (1.384, 2.143) | 1.39E-06 | 0.086 | 0.955 | 0.499844 | 2q24.2 | 159778002 |
| *ZNF385B* | *rs16866933* | *1.45E-15* | *1.54E-10* | *1.69 (1.475, 1.925)* | *6.15E-14* | *0.085* | *0.977* | *0.059204* | *2q31.2* | *180274923* |
| TMEFF2 | rs13007495 | 3.90E-07 | 7.12E-05 | 1.47 (1.248, 1.73) | 4.49E-06 | 0.144 | 0.967 | 0.067067 | 2q32.3 | 192663750 |
|  | rs2253615 | 3.03E-05 | 4.47E-04 | 1.4 (1.174, 1.666) | 1.82E-04 | 0.151 | 0.998 | 0.048155 | 2q35 | 220477123 |
| SPHKAP | rs7577229 | 2.43E-05 | 9.85E-04 | 1.44 (1.193, 1.74) | 1.62E-04 | 0.131 | 1.000 | 0.209214 | 2q36.3 | 228699882 |
|  | rs12052730 | 4.72E-06 | 2.08E-04 | 1.42 (1.179, 1.699) | 2.10E-04 | 0.142 | 1.000 | 0.110912 | 2q36.3 | 228896256 |
| NGEF | rs895431 | 4.33E-05 | 5.43E-04 | 1.18 (1.085, 1.287) | 1.33E-04 | 0.313 | 0.997 | 0.015942 | 2q37.1 | 233467606 |
| NGEF | rs778362 | 4.69E-05 | 5.66E-04 | 1.24 (1.099, 1.389) | 4.19E-04 | 0.208 | 0.997 | 0.217860 | 2q37.1 | 233488430 |
| NGEF | rs3811588 | 1.56E-05 | 9.02E-04 | 1.64 (1.311, 2.05) | 1.66E-05 | 0.105 | 0.993 | 0.209154 | 2q37.1 | 233584219 |
| CHL1 | rs6764363 | 6.61E-08 | 1.85E-06 | 1.19 (1.104, 1.282) | 6.31E-06 | 0.356 | 1.000 | 0.010621 | 3p26.3 | 287349 |
| CADPS | rs833637 | 5.14E-06 | 3.91E-05 | 1.2 (1.102, 1.315) | 4.14E-05 | 0.269 | 0.998 | 0.815310 | 3p14.2 | 62558220 |
| CADPS | rs17067111 | 2.58E-05 | 2.90E-03 | 2.07 (1.472, 2.915) | 3.23E-05 | 0.060 | 1.000 | 0.244568 | 3p14.2 | 62697011 |
|  | rs7643940 | 1.60E-05 | 2.19E-04 | 1.3 (1.147, 1.478) | 5.00E-05 | 0.146 | 0.980 | 0.461485 | 3p14.1 | 70495866 |
|  | rs16849176 | 2.98E-05 | 1.80E-03 | 1.61 (1.236, 2.109) | 4.55E-04 | 0.085 | 1.000 | 0.351551 | 3q13.33 | 121845011 |
| *HEG1* | *rs1574733* | *8.01E-05* | *2.27E-03* | *1.52 (1.235, 1.882)* | *9.52E-05* | *0.128* | *0.992* | *0.090694* | *3q21.2* | *126240008* |
|  | rs17081601 | 3.31E-05 | 8.43E-05 | 1.45 (1.213, 1.726) | 4.45E-05 | 0.078 | 0.993 | 0.083238 | 4p15.1 | 32230595 |
|  | rs1397933 | 3.76E-06 | 9.23E-05 | 1.23 (1.114, 1.362) | 5.44E-05 | 0.210 | 0.960 | 0.891611 | 4p14 | 39863932 |
|  | rs4956081 | 4.69E-05 | 2.29E-06 | 0.89 (0.836, 0.94) | 6.52E-05 | 0.459 | 0.995 | 0.427266 | 4q25 | 108384331 |
| ARSJ | rs6834503 | 2.48E-05 | 9.30E-05 | 1.11 (1.054, 1.177) | 1.42E-04 | 0.499 | 1.000 | 1.000000 | 4q26 | 114990624 |
|  | rs2000601 | 7.38E-05 | 4.54E-04 | 1.19 (1.086, 1.298) | 1.80E-04 | 0.265 | 0.995 | 0.812812 | 4q26 | 116318715 |
|  | rs7694946 | 1.67E-05 | 1.47E-04 | 1.21 (1.108, 1.326) | 2.89E-05 | 0.261 | 0.969 | 0.714534 | 4q26 | 116413588 |
|  | rs4834522 | 4.82E-06 | 2.00E-04 | 1.36 (1.158, 1.598) | 1.88E-04 | 0.144 | 0.998 | 0.336095 | 4q26 | 117407102 |
|  | rs17688098 | 4.12E-08 | 3.76E-06 | 1.34 (1.176, 1.535) | 1.63E-05 | 0.156 | 0.990 | 0.719451 | 4q26 | 117446883 |
|  | rs2389202 | 2.86E-09 | 8.16E-07 | 1.43 (1.249, 1.648) | 4.38E-07 | 0.128 | 0.967 | 0.642674 | 4q26 | 117473738 |
|  | rs7685403 | 2.20E-06 | 5.54E-06 | 1.25 (1.12, 1.401) | 8.60E-05 | 0.161 | 1.000 | 0.033418 | 4q26 | 117479188 |
|  | rs1489014 | 1.11E-07 | 1.72E-05 | 1.36 (1.178, 1.575) | 3.47E-05 | 0.137 | 0.990 | 1.000000 | 4q26 | 117491906 |
| *SPRY1* | *rs159864* | *1.15E-06* | *4.14E-04* | *1.82 (1.347, 2.449)* | *9.85E-05* | *0.094* | *0.995* | *0.024898* | *4q28.1* | *124516537* |
|  | rs1036342 | 7.48E-05 | 1.86E-04 | 1.21 (1.101, 1.324) | 6.64E-05 | 0.276 | 0.997 | 0.141143 | 4q32.1 | 158712424 |
| GRIA2 | rs1352693 | 3.41E-05 | 1.01E-03 | 1.48 (1.226, 1.789) | 5.15E-05 | 0.136 | 0.995 | 0.078865 | 4q32.1 | 158811013 |
| FSTL5 | rs12512615 | 2.53E-05 | 2.92E-03 | 1.88 (1.331, 2.653) | 3.51E-04 | 0.066 | 0.987 | 0.400052 | 4q32.2 | 163268047 |
|  | rs6824106 | 6.51E-05 | 3.24E-04 | 1.16 (1.075, 1.241) | 9.27E-05 | 0.372 | 0.987 | 0.054150 | 4q34.3 | 179038012 |
|  | rs12650550 | 4.74E-06 | 4.05E-04 | 1.8 (1.308, 2.464) | 3.13E-04 | 0.061 | 1.000 | 0.244349 | 4q35.1 | 185478310 |
|  | rs6839415 | 6.86E-06 | 2.13E-04 | 1.44 (1.196, 1.728) | 1.18E-04 | 0.060 | 0.985 | 0.063573 | 4q35.2 | 187474299 |
| FAT | rs397321 | 7.76E-05 | 6.59E-04 | 1.33 (1.146, 1.551) | 2.09E-04 | 0.158 | 1.000 | 0.472137 | 4q35.2 | 188052641 |
|  | rs7708079 | 3.96E-05 | 2.90E-03 | 1.88 (1.333, 2.657) | 3.44E-04 | 0.057 | 0.988 | 0.387063 | 5p13.1 | 39684321 |
|  | rs10059203 | 1.79E-06 | 1.58E-05 | 1.14 (1.073, 1.221) | 4.92E-05 | 0.413 | 0.995 | 0.265140 | 5p13.1 | 40087842 |
| SNX18 | rs17442098 | 8.46E-05 | 2.19E-03 | 1.55 (1.259, 1.916) | 4.27E-05 | 0.106 | 1.000 | 0.342329 | 5q11.2 | 53908159 |
|  | rs292201 | 4.75E-05 | 6.82E-04 | 1.38 (1.188, 1.603) | 2.72E-05 | 0.158 | 0.995 | 0.296984 | 5q13.3 | 73626850 |
|  | rs294986 | 8.39E-05 | 8.18E-04 | 1.32 (1.164, 1.488) | 1.29E-05 | 0.190 | 1.000 | 0.379948 | 5q13.3 | 73718795 |
| ENC1 | rs467147 | 7.27E-05 | 2.19E-03 | 1.53 (1.241, 1.891) | 7.68E-05 | 0.091 | 1.000 | 0.786061 | 5q13.3 | 73747302 |
| CETN3 | rs6452846 | 1.59E-05 | 2.42E-04 | 1.25 (1.111, 1.416) | 2.69E-04 | 0.185 | 1.000 | 1.000000 | 5q14.3 | 89040983 |
|  | rs4621553 | 2.18E-08 | 5.49E-06 | 1.4 (1.243, 1.578) | 4.12E-08 | 0.220 | 0.995 | 0.001665 | 5q22.2 | 113058063 |
|  | rs1871912 | 1.70E-05 | 2.42E-04 | 1.25 (1.122, 1.387) | 4.56E-05 | 0.228 | 1.000 | 0.249019 | 5q22.2 | 113082398 |
| *FTMT* | *rs4305661* | *2.93E-05* | *1.79E-03* | *1.73 (1.33, 2.258)* | *5.22E-05* | *0.097* | *0.990* | *0.163630* | *5q23.1* | *120512682* |
|  | rs4835938 | 2.43E-06 | 1.01E-04 | 1.3 (1.141, 1.471) | 7.11E-05 | 0.131 | 0.969 | 0.156482 | 5q23.3 | 127404930 |
| PCDH1 | rs152274 | 4.94E-05 | 7.63E-05 | 1.15 (1.07, 1.23) | 1.30E-04 | 0.368 | 0.998 | 0.627596 | 5q31.3 | 141135339 |
| GRIA1 | rs12189362 | 2.23E-12 | 9.94E-09 | 1.5 (1.322, 1.693) | 3.22E-10 | 0.117 | 0.977 | 0.242562 | 5q33.2 | 153037741 |
|  | rs6917581 | 1.29E-05 | 9.47E-04 | 1.57 (1.251, 1.963) | 1.01E-04 | 0.121 | 0.995 | 0.031299 | 6p25.3 | 1002990 |
| FARS2 | rs1009606 | 4.30E-05 | 1.80E-03 | 1.61 (1.236, 2.108) | 4.60E-04 | 0.053 | 1.000 | 1.000000 | 6p25.1 | 5671368 |
| GPSM3 | rs424232 | 3.33E-05 | 1.25E-04 | 1.23 (1.111, 1.368) | 8.57E-05 | 0.266 | 0.988 | 0.012722 | 6p21.32 | 32316302 |
| PKHD1 | rs9296658 | 3.63E-06 | 6.00E-05 | 1.18 (1.077, 1.294) | 3.93E-04 | 0.271 | 1.000 | 0.250670 | 6p12.2 | 51597907 |
| *COL21A1* | *rs17817377* | *2.38E-05* | *2.92E-03* | *2.01 (1.424, 2.824)* | *7.44E-05* | *0.076* | *0.998* | *0.097892* | *6p12.1* | *56024937* |
| *ESR1* | *rs2982694* | *3.91E-12* | *2.62E-08* | *1.43 (1.277, 1.596)* | *6.96E-10* | *0.138* | *0.975* | *0.160104* | *6q25.1* | *152327380* |
|  | rs732577 | 1.52E-05 | 7.81E-05 | 1.15 (1.083, 1.217) | 3.74E-06 | 0.496 | 0.977 | 0.019903 | 7p21.3 | 12898574 |
| *ADCYAP1R1* | *rs17159861* | *2.81E-05* | *1.83E-03* | *1.79 (1.375, 2.335)* | *1.76E-05* | *0.115* | *0.997* | *0.008174* | *7p15.1* | *31051687* |
| POU6F2 | rs13246263 | 4.90E-05 | 7.79E-04 | 1.29 (1.125, 1.474) | 2.50E-04 | 0.179 | 1.000 | 0.343377 | 7p14.1 | 39290995 |
|  | rs6964415 | 5.24E-09 | 2.16E-06 | 1.42 (1.241, 1.626) | 4.73E-07 | 0.081 | 0.975 | 0.001644 | 7p13 | 46209108 |
| *FZD1* | *rs1076464* | *9.54E-05* | *4.74E-04* | *1.19 (1.081, 1.301)* | *3.35E-04* | *0.279* | *0.993* | *0.091398* | *7q21.2* | *91024410* |
| SAMD9 | rs1123915 | 1.57E-05 | 9.25E-04 | 1.64 (1.308, 2.048) | 1.86E-05 | 0.118 | 1.000 | 0.046824 | 7q21.2 | 92503820 |
| OCM2 | rs13438327 | 8.06E-07 | 4.05E-04 | 2.05 (1.527, 2.756) | 2.19E-06 | 0.089 | 1.000 | 0.039322 | 7q21.3 | 97458627 |
|  | rs193795 | 8.04E-05 | 1.69E-05 | 0.87 (0.81, 0.927) | 3.81E-05 | 0.417 | 0.954 | 0.581752 | 7q22.2 | 105408748 |
| ***NOS3*** | ***rs10264084*** | ***3.07E-05*** | ***4.86E-04*** | ***1.35 (1.172, 1.543)*** | ***2.63E-05*** | ***0.171*** | ***1.000*** | ***0.329690*** | ***7q36.1*** | ***150223295*** |
|  | rs17718923 | 6.42E-06 | 3.25E-04 | 1.36 (1.17, 1.591) | 8.04E-05 | 0.110 | 0.983 | 0.479095 | 7q36.3 | 156676187 |
|  | rs10112533 | 4.10E-06 | 1.07E-04 | 1.25 (1.114, 1.408) | 1.76E-04 | 0.229 | 1.000 | 0.013977 | 8p23.3 | 283820 |
| CSMD1 | rs17761209 | 2.38E-05 | 3.99E-04 | 1.37 (1.151, 1.633) | 4.28E-04 | 0.113 | 0.980 | 1.000000 | 8p23.2 | 2653888 |
| EFCAB1 | rs10087659 | 6.35E-05 | 3.10E-04 | 1.38 (1.185, 1.618) | 4.74E-05 | 0.148 | 0.990 | 0.279478 | 8q11.21 | 49982983 |
| C8orf34 | rs7838605 | 2.73E-05 | 2.90E-03 | 1.86 (1.32, 2.625) | 4.18E-04 | 0.065 | 1.000 | 0.244349 | 8q13.2 | 69829443 |
| PXMP3 | rs3864663 | 3.15E-06 | 1.82E-04 | 1.4 (1.222, 1.61) | 1.87E-06 | 0.145 | 0.985 | 0.596800 | 8q21.12 | 78842904 |
| PGCP | rs2874113 | 2.47E-05 | 5.24E-05 | 1.18 (1.088, 1.271) | 4.77E-05 | 0.354 | 0.995 | 0.010185 | 8q22.1 | 97816301 |
| *ZFPM2* | *rs16872085* | *1.18E-06* | *2.91E-04* | *1.83 (1.439, 2.328)* | *1.03E-06* | *0.085* | *0.993* | *0.348833* | *8q22.3* | *106026716* |
| KCNK9 | rs4736057 | 2.45E-06 | 1.05E-04 | 1.29 (1.128, 1.477) | 2.14E-04 | 0.096 | 0.974 | 0.000812 | 8q24.3 | 140197024 |
| NFIB | rs717932 | 4.17E-06 | 1.18E-05 | 1.14 (1.069, 1.206) | 4.18E-05 | 0.474 | 0.988 | 0.004281 | 9p22.3 | 14379011 |
|  | rs2498432 | 1.87E-05 | 2.90E-03 | 1.89 (1.341, 2.666) | 2.97E-04 | 0.091 | 1.000 | 0.023961 | 9q21.11 | 70874189 |
|  | rs16907764 | 9.51E-05 | 2.92E-03 | 2.05 (1.425, 2.943) | 1.16E-04 | 0.063 | 0.998 | 0.244497 | 9q22.31 | 93574738 |
|  | rs1954599 | 2.72E-05 | 1.91E-03 | 1.69 (1.294, 2.209) | 1.26E-04 | 0.089 | 0.985 | 0.236677 | 9q22.31 | 93769594 |
|  | rs10992675 | 8.60E-05 | 1.80E-03 | 1.66 (1.259, 2.183) | 3.35E-04 | 0.081 | 1.000 | 0.346100 | 9q22.31 | 95003137 |
| CORO2A | rs10985391 | 2.18E-05 | 9.18E-04 | 1.41 (1.164, 1.701) | 4.42E-04 | 0.097 | 0.965 | 1.000000 | 9q22.33 | 100000428 |
|  | rs10990394 | 2.08E-05 | 2.79E-03 | 1.95 (1.384, 2.739) | 1.40E-04 | 0.115 | 0.990 | 0.002258 | 9q31.1 | 104765087 |
| ASS1 | rs7860909 | 2.13E-05 | 1.69E-04 | 1.19 (1.098, 1.286) | 2.16E-05 | 0.341 | 0.990 | 0.015593 | 9q34.11 | 132321047 |
| USP6NL | rs17150540 | 3.42E-05 | 2.90E-03 | 2.04 (1.451, 2.877) | 4.72E-05 | 0.057 | 1.000 | 0.386698 | 10p14 | 11620360 |
|  | rs10829156 | 2.08E-10 | 9.28E-08 | 1.28 (1.164, 1.406) | 4.41E-07 | 0.205 | 0.965 | 0.477866 | 10p12.33 | 18990561 |
| *PLXDC2* | *rs12358819* | *3.50E-05* | *8.91E-04* | *1.43 (1.207, 1.683)* | *3.28E-05* | *0.139* | *0.992* | *0.430961* | *10p12.31* | *20817949* |
| PRKG1 | rs4935303 | 7.43E-05 | 1.54E-03 | 1.39 (1.161, 1.661) | 3.49E-04 | 0.161 | 0.980 | 0.023597 | 10q21.1 | 53439614 |
| ANK3 | rs12269538 | 3.13E-05 | 1.65E-03 | 1.46 (1.185, 1.796) | 3.91E-04 | 0.088 | 0.975 | 1.000000 | 10q21.2 | 61950242 |
| *ARID5B* | *rs9415637* | *1.17E-07* | *3.09E-05* | *1.55 (1.27, 1.896)* | *1.91E-05* | *0.109* | *0.992* | *0.208322* | *10q21.2* | *63510271* |
| COL13A1 | rs12357925 | 6.74E-05 | 5.64E-04 | 1.27 (1.127, 1.422) | 7.89E-05 | 0.190 | 0.985 | 1.000000 | 10q21.3 | 71279319 |
| *NRG3* | *rs6584391* | *1.98E-05* | *1.83E-03* | *1.68 (1.288, 2.193)* | *1.41E-04* | *0.062* | *0.997* | *1.000000* | *10q23.1* | *83643718* |
| PLCE1 | rs11187837 | 8.37E-08 | 5.51E-05 | 1.79 (1.431, 2.236) | 4.22E-07 | 0.119 | 0.964 | 0.019426 | 10q23.33 | 96025970 |
| *HTRA1* | *rs2247541* | *2.06E-05* | *4.54E-04* | *1.48 (1.206, 1.812)* | *1.78E-04* | *0.109* | *0.985* | *0.456715* | *10q26.13* | *124250729* |
|  | rs1875775 | 2.27E-05 | 1.81E-03 | 1.66 (1.273, 2.165) | 1.97E-04 | 0.095 | 0.987 | 0.236493 | 11p15.3 | 11129277 |
| NAV2 | rs7106863 | 4.48E-05 | 6.66E-04 | 1.33 (1.145, 1.546) | 2.06E-04 | 0.157 | 0.998 | 0.377936 | 11p15.1 | 19876485 |
|  | rs10833905 | 6.96E-07 | 2.07E-05 | 1.32 (1.183, 1.469) | 6.81E-07 | 0.219 | 1.000 | 0.278169 | 11p14.3 | 23001168 |
|  | rs4076032 | 1.08E-05 | 6.69E-04 | 1.48 (1.23, 1.778) | 3.45E-05 | 0.089 | 0.972 | 0.773623 | 11p12 | 42555769 |
|  | rs10765792 | 5.69E-15 | 4.89E-10 | 1.54 (1.369, 1.724) | 8.47E-13 | 0.111 | 0.980 | 0.031706 | 11q21 | 95506348 |
| *ZBTB16* | *rs17116384* | *4.24E-05* | *2.86E-04* | *1.18 (1.083, 1.289)* | *1.77E-04* | *0.308* | *0.972* | *0.032476* | *11q23.2* | *113486451* |
| *ZBTB16* | *rs238913* | *5.13E-05* | *1.21E-04* | *1.15 (1.075, 1.239)* | *8.05E-05* | *0.377* | *1.000* | *0.045453* | *11q23.2* | *113489699* |
|  | rs1894063 | 2.79E-05 | 2.90E-03 | 1.99 (1.415, 2.807) | 8.53E-05 | 0.064 | 1.000 | 0.247048 | 11q23.3 | 115505004 |
| *ETS1* | *rs2217042* | *9.60E-06* | *8.66E-04* | *1.55 (1.24, 1.945)* | *1.37E-04* | *0.106* | *0.990* | *0.209248* | *11q24.2* | *127387080* |
| B3GAT1 | rs10791373 | 2.60E-06 | 1.64E-04 | 1.32 (1.144, 1.533) | 1.81E-04 | 0.171 | 1.000 | 0.135844 | 11q25 | 133853184 |
|  | rs11604608 | 1.91E-05 | 2.77E-03 | 1.95 (1.387, 2.733) | 1.25E-04 | 0.056 | 0.980 | 0.387174 | 11q25 | 134108156 |
| ***CACNA1C*** | ***rs7132154*** | ***9.29E-05*** | ***4.72E-04*** | ***1.18 (1.079, 1.29)*** | ***3.09E-04*** | ***0.262*** | ***0.992*** | ***1.000000*** | ***12p13.33*** | ***2331484*** |
| *C1S* | *rs12146727* | *9.01E-05* | *1.92E-03* | *1.46 (1.222, 1.75)* | *3.63E-05* | *0.131* | *1.000* | *0.311476* | *12p13.31* | *7040597* |
| *CPNE8* | *rs12231816* | *2.51E-05* | *2.81E-03* | *1.85 (1.317, 2.607)* | *4.14E-04* | *0.066* | *0.998* | *0.245177* | *12q12* | *37811403* |
| ATF1 | rs17291650 | 4.88E-07 | 5.62E-05 | 1.43 (1.248, 1.637) | 3.10E-07 | 0.103 | 0.992 | 0.039692 | 12q13.13 | 49499700 |
| KCNC2 | rs17114532 | 9.22E-05 | 2.19E-03 | 1.47 (1.19, 1.814) | 3.70E-04 | 0.129 | 1.000 | 0.090403 | 12q21.1 | 73737645 |
|  | rs7307780 | 2.27E-16 | 3.11E-12 | 1.45 (1.327, 1.592) | 5.02E-15 | 0.186 | 0.960 | 0.141220 | 12q21.2 | 74506885 |
|  | rs1499928 | 6.62E-05 | 1.10E-03 | 1.35 (1.169, 1.562) | 5.25E-05 | 0.168 | 1.000 | 0.247316 | 12q24.33 | 129031885 |
| PARP4 | rs9581094 | 6.47E-07 | 1.68E-04 | 1.7 (1.383, 2.1) | 7.06E-07 | 0.151 | 1.000 | 0.000375 | 13q12.12 | 23980630 |
| P2RY5 | rs2227311 | 2.58E-05 | 9.85E-04 | 1.5 (1.244, 1.812) | 2.50E-05 | 0.132 | 1.000 | 0.210531 | 13q14.2 | 47885033 |
| KLF12 | rs1886452 | 2.05E-05 | 8.63E-05 | 1.17 (1.075, 1.269) | 2.51E-04 | 0.296 | 0.992 | 1.000000 | 13q22.1 | 72884855 |
|  | rs12429889 | 8.93E-23 | 7.08E-16 | 1.64 (1.479, 1.812) | 5.27E-20 | 0.161 | 0.954 | 1.000000 | 13q22.1 | 73640323 |
|  | rs2151866 | 2.48E-05 | 2.90E-03 | 2.03 (1.445, 2.863) | 5.15E-05 | 0.103 | 1.000 | 0.005883 | 13q31.1 | 81010242 |
| AP1G2 | rs2281680 | 5.45E-06 | 1.54E-04 | 1.38 (1.227, 1.542) | 6.05E-08 | 0.212 | 0.982 | 0.260456 | 14q11.2 | 23102910 |
| LRFN5 | rs4904757 | 6.46E-05 | 2.84E-03 | 1.91 (1.334, 2.733) | 4.30E-04 | 0.081 | 0.995 | 0.061324 | 14q21.2 | 41274666 |
|  | rs11624056 | 8.59E-10 | 1.20E-06 | 1.43 (1.26, 1.615) | 3.00E-08 | 0.110 | 0.967 | 0.028261 | 14q31.3 | 86576001 |
| OCA2 | rs11857677 | 2.71E-05 | 2.90E-03 | 1.94 (1.38, 2.739) | 1.54E-04 | 0.072 | 1.000 | 0.154442 | 15q13.1 | 25973203 |
| AQR | rs11852984 | 9.77E-06 | 1.62E-04 | 1.24 (1.109, 1.397) | 2.23E-04 | 0.206 | 0.990 | 0.477507 | 15q14 | 32892949 |
| SIN3A | rs8028182 | 1.47E-06 | 5.58E-05 | 1.36 (1.194, 1.541) | 3.49E-06 | 0.184 | 1.000 | 0.516361 | 15q24.2 | 73505722 |
| TMC3 | rs9630440 | 1.73E-05 | 2.86E-03 | 1.98 (1.406, 2.777) | 9.53E-05 | 0.061 | 0.993 | 0.394456 | 15q25.1 | 79429119 |
| MCTP2 | rs1014922 | 4.88E-07 | 5.24E-06 | 1.36 (1.197, 1.552) | 3.44E-06 | 0.101 | 0.983 | 0.008130 | 15q26.2 | 92966584 |
| C16orf68 | rs8061218 | 3.06E-05 | 3.05E-04 | 1.76 (1.362, 2.284) | 1.89E-05 | 0.087 | 0.995 | 0.352363 | 16p13.2 | 7782577 |
| ABAT | rs8049072 | 9.26E-05 | 2.19E-03 | 1.46 (1.185, 1.807) | 4.27E-04 | 0.109 | 1.000 | 0.461151 | 16p13.2 | 8769713 |
|  | rs17550532 | 4.06E-06 | 1.07E-04 | 1.33 (1.181, 1.489) | 2.14E-06 | 0.212 | 0.988 | 0.166063 | 16p13.2 | 9651596 |
|  | rs12596791 | 2.96E-05 | 2.46E-04 | 1.18 (1.078, 1.295) | 3.63E-04 | 0.260 | 0.998 | 0.639608 | 16p12.1 | 26023063 |
| CDH8 | rs12599168 | 2.58E-05 | 2.81E-03 | 1.86 (1.321, 2.62) | 3.96E-04 | 0.084 | 0.998 | 0.100234 | 16q21 | 60509210 |
| CDH13 | rs4782812 | 2.30E-05 | 2.68E-01 | 0.04 (0.01, 0.186) | 2.34E-05 | 0.061 | 0.998 | 1.000000 | 16q23.3 | 82121112 |
|  | rs11645915 | 2.16E-05 | 1.80E-03 | 1.79 (1.372, 2.329) | 1.90E-05 | 0.115 | 1.000 | 0.019925 | 16q24.1 | 84849794 |
| GARNL4 | rs12603284 | 1.33E-07 | 5.79E-05 | 1.89 (1.451, 2.47) | 3.07E-06 | 0.079 | 0.970 | 0.160705 | 17p13.3 | 2718262 |
|  | rs16954697 | 1.02E-06 | 1.14E-04 | 1.43 (1.178, 1.732) | 3.05E-04 | 0.135 | 0.959 | 0.062346 | 17p13.2 | 5297637 |
|  | rs4356515 | 2.32E-05 | 2.94E-04 | 1.25 (1.134, 1.388) | 1.29E-05 | 0.208 | 0.987 | 1.000000 | 17p12 | 15029018 |
| TNFRSF13B | rs6502546 | 4.44E-05 | 1.85E-04 | 1.13 (1.059, 1.203) | 2.07E-04 | 0.426 | 1.000 | 0.083396 | 17p11.2 | 16819213 |
| *ACCN1* | *rs7226216* | *8.64E-05* | *2.22E-03* | *1.47 (1.188, 1.813)* | *3.88E-04* | *0.130* | *0.997* | *0.091685* | *17q11.2* | *28354024* |
| KRT13 | rs903 | 5.14E-05 | 3.51E-04 | 1.14 (1.066, 1.21) | 8.46E-05 | 0.409 | 0.987 | 0.852959 | 17q21.2 | 36910863 |
|  | rs3809758 | 1.37E-05 | 2.94E-04 | 1.33 (1.158, 1.537) | 7.23E-05 | 0.184 | 0.998 | 0.084308 | 17q21.2 | 37725506 |
|  | rs17718586 | 1.70E-08 | 7.26E-06 | 1.53 (1.324, 1.775) | 1.70E-08 | 0.101 | 0.964 | 0.424883 | 17q24.3 | 66155784 |
|  | rs1347545 | 5.56E-05 | 3.77E-04 | 1.16 (1.079, 1.257) | 9.32E-05 | 0.322 | 0.987 | 1.000000 | 17q25.2 | 72832035 |
| C18orf2 | rs789046 | 3.33E-05 | 2.90E-03 | 1.92 (1.359, 2.7) | 2.18E-04 | 0.093 | 1.000 | 0.038384 | 18p11.32 | 1010820 |
|  | rs597503 | 1.49E-11 | 1.33E-07 | 1.45 (1.273, 1.646) | 2.34E-08 | 0.127 | 0.959 | 0.668482 | 18p11.31 | 6929947 |
| PTPRM | rs16953201 | 3.04E-05 | 6.35E-04 | 1.31 (1.146, 1.495) | 7.58E-05 | 0.182 | 0.978 | 0.423041 | 18p11.23 | 8288073 |
| *RAB12* | *rs12185468* | *1.53E-05* | *9.01E-04* | *1.5 (1.196, 1.88)* | *4.62E-04* | *0.113* | *0.970* | *0.145045* | *18p11.23* | *8469685* |
| IMPA2 | rs2002212 | 4.19E-05 | 2.84E-03 | 1.98 (1.409, 2.796) | 9.60E-05 | 0.063 | 0.995 | 0.250337 | 18p11.21 | 11996035 |
| KCTD1 | rs16942421 | 9.11E-11 | 3.33E-07 | 1.68 (1.429, 1.981) | 7.75E-10 | 0.081 | 0.975 | 0.742069 | 18q11.2 | 22410423 |
|  | rs12957518 | 9.28E-05 | 2.38E-03 | 1.48 (1.199, 1.836) | 2.94E-04 | 0.112 | 0.982 | 0.339216 | 18q12.2 | 33779731 |
| C18orf26 | rs2008315 | 2.62E-06 | 2.00E-04 | 1.39 (1.182, 1.629) | 6.72E-05 | 0.157 | 0.998 | 0.109759 | 18q21.2 | 50635887 |
|  | rs12456161 | 2.45E-06 | 1.58E-04 | 1.38 (1.196, 1.596) | 1.31E-05 | 0.181 | 0.993 | 0.020886 | 18q21.31 | 53311687 |
| OR1I1 | rs12975625 | 1.74E-05 | 2.90E-03 | 1.87 (1.326, 2.638) | 3.79E-04 | 0.079 | 1.000 | 0.157797 | 19p13.12 | 15059764 |
| RSHL1 | rs8111071 | 9.20E-07 | 4.05E-04 | 2.01 (1.495, 2.7) | 4.34E-06 | 0.088 | 1.000 | 0.040471 | 19q13.32 | 50999246 |
| *MZF1* | *rs3794963* | *3.03E-05* | *4.84E-05* | *1.21 (1.095, 1.335)* | *1.75E-04* | *0.247* | *0.985* | *0.619667* | *19q13.43* | *63762863* |
| PTPRT | rs11696306 | 4.70E-07 | 1.77E-05 | 1.24 (1.125, 1.356) | 1.04E-05 | 0.211 | 0.975 | 0.220466 | 20q12 | 40359539 |
| WFDC2 | rs16990209 | 3.21E-05 | 1.90E-03 | 1.69 (1.291, 2.203) | 1.38E-04 | 0.080 | 0.987 | 0.498667 | 20q13.12 | 43527964 |
|  | rs5762311 | 3.57E-06 | 4.08E-04 | 2.03 (1.494, 2.753) | 6.75E-06 | 0.089 | 0.987 | 0.038357 | 22q12.1 | 26420207 |
| MN1 | rs4820774 | 8.06E-05 | 1.37E-04 | 1.17 (1.078, 1.267) | 1.63E-04 | 0.306 | 0.997 | 0.595357 | 22q12.1 | 26432894 |
|  | rs54211 | 9.09E-05 | 1.92E-03 | 1.57 (1.317, 1.881) | 7.72E-07 | 0.132 | 1.000 | 0.311653 | 22q13.1 | 38017430 |
