# Supplementary material for: GWAS for discovery and replication of genetic loci associated with sudden cardiac arrest in patients with coronary artery disease
Source: BMC Cardiovasc Disord. 2011 Jun 10;11:29. doi: 10.1186/1471-2261-11-29 (PMC3141757; doi:10.1186/1471-2261-11-29)

### Additional file 6: Supplemental Figure 5

**Title: GWAS Q-Q plots featuring location of candidate SNPs**

# Description: Q-Q plots of the negative log10 P values for PCA-corrected correlation tests for genome-wide association across the genome are shown for the additive (panel A), Dominant (panel B), and Recessive (panel C) models. Those SNPs associations that reached genome-wide significant (Bonferroni-p <0.05) are colored in red.

### A]


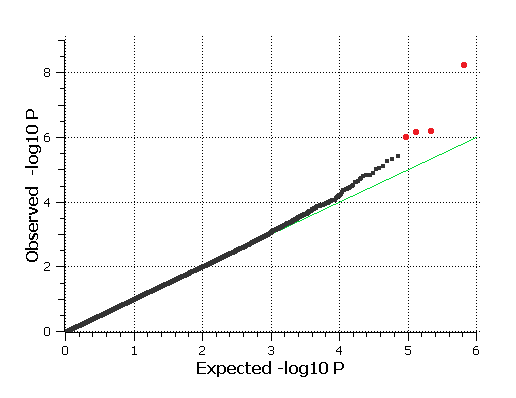


**B]**


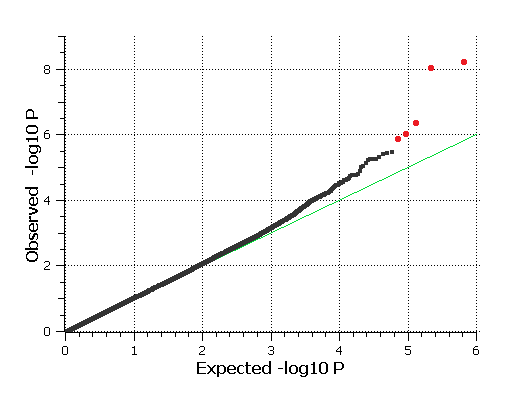


**C]**


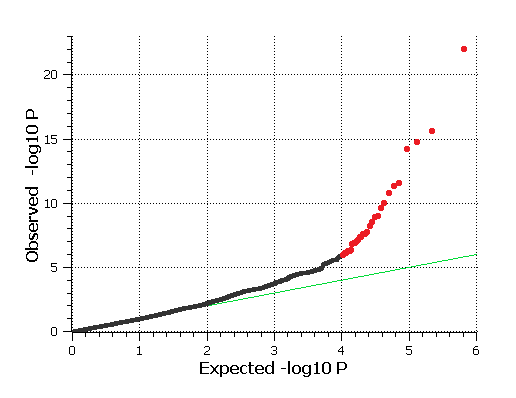

Supplement: Additional file 13 — GWAS Q-Q plots featuring location of candidate SNPs. Q-Q plots of the negative log10 P values for PCA-corrected correlation tests for genome-wide association across the genome are shown for the additive (panel A), Dominant (panel B), and Recessive (panel C) models. Those SNPs associations that reached genome-wide significant (Bonferroni-p <0.05) are colored in red. [file 1471-2261-11-29-S13.DOC]
